# Histological assessment of the intramural segment in adults with an anomalous aortic origin of a coronary artery with an interarterial course

**DOI:** 10.1186/s12872-026-06000-7

**Published:** 2026-06-19

**Authors:** Diederick B.H. Verheijen, Tamara C. Borsboom, Philippine Kiès, Anastasia D. Egorova, Lambertus J. Wisse, Dave R. Koolbergen, Léon M. Putman, Mark G. Hazekamp, Roel L.F. van der Palen, J. Wouter Jukema, Ryan E. Accord, Hubert W. Vliegen, Marco C. de Ruiter, Monique R.M. Jongbloed

**Affiliations:** 1 Center for Congenital Heart Disease Amsterdam-Leiden (CAHAL), Leiden, the Netherlands; 2 Department of Cardiology, Leiden University Medical Center, Leiden, the Netherlands; 3 Department of Anatomy and Embryology, Leiden University Medical Center, Leiden, the Netherlands; 4 Department of Cardiothoracic surgery, Leiden University Medical Center, Leiden, the Netherlands; 5 Division of Pediatric Cardiology, Department of Pediatrics, Leiden University Medical Center, Leiden, the Netherlands; 6 Netherlands Heart Institute, Utrecht, The Netherlands; 7 Department of Cardiothoracic Surgery, University Medical Center Groningen, Groningen, the Netherlands

**Keywords:** Anomalous aortic origin of the coronary artery, Interarterial course, Intramural segment, Intramural course, Histology, Tunica media

## Abstract

**Background:**

An intramural course in an anomalous aortic origin of a coronary artery (AAOCA) is a high-risk feature for sudden cardiac death (SCD). One of the proposed mechanisms for this association involves a “flap valve” effect in the intramural segment. The (patho-)histological characteristics of this segment may contribute to risk stratification, however, current data are scarce. The aim of this study was to identify the histological features of the intramural segment and their potential relevance to vascular wall properties and the aortic valve commissure, thereby providing insights into possible functional implications of AAOCA.

**Methods:**

This prospective multicenter study included consecutive pediatric and adult AAOCA patients who underwent surgical unroofing between 2021 and 2024. The excised arterial vascular wall tissue (i.e. the intramural segment), was immunohistochemically examined for vascular wall components. Two independent observers reviewed the data, correlating findings with preoperative CTA to assess spatial relationships with surrounding aortic and AAOCA tissues. Results were also compared with a post-mortem AAOCA specimen and control tissues.

**Results:**

Fifteen patients (mean age 42.9 ± 14.0 years, age range 11–66 years, 60% female) undergoing surgery for AAOCA were included. Fourteen patients (93%) had an anomalous aortic origin of a right coronary artery (AAORCA) and one patient (7%) had an anomalous aortic origin of a left coronary artery (AAOLCA). The mean intramural segment length was 7.8 ± 3.6 mm in AAORCA and 16.0 mm in the AAOLCA. Histologically, a two-layered tunica media without interposing tunica adventitia was identified in all intramural segments. Pre-operative CTA showed a close spatial relationship of the aortic side of the intramural segment with the aortic valve commissure. In line with this, extensive fibrous tissue was observed histologically on the aortic side of the intramural segment, consistent with commissure. In contrast, the vascular wall on the coronary arterial side of the intramural segment showed overall medial degeneration, characterized by mucoid extracellular matrix accumulation (MEMA), elastic fiber fragmentation and/or loss, elastic fiber thinning, loss of smooth muscle cell nuclei, and collagen alterations. These abnormalities were more prevalent with increasing age.

**Conclusion:**

Histology of the intramural segment revealed structural alterations which may be consistent with reduced vascular compliance. Further studies, including post-SCD specimens, are needed to refine risk stratification.

**Graphical Abstract:**

**Figure:**
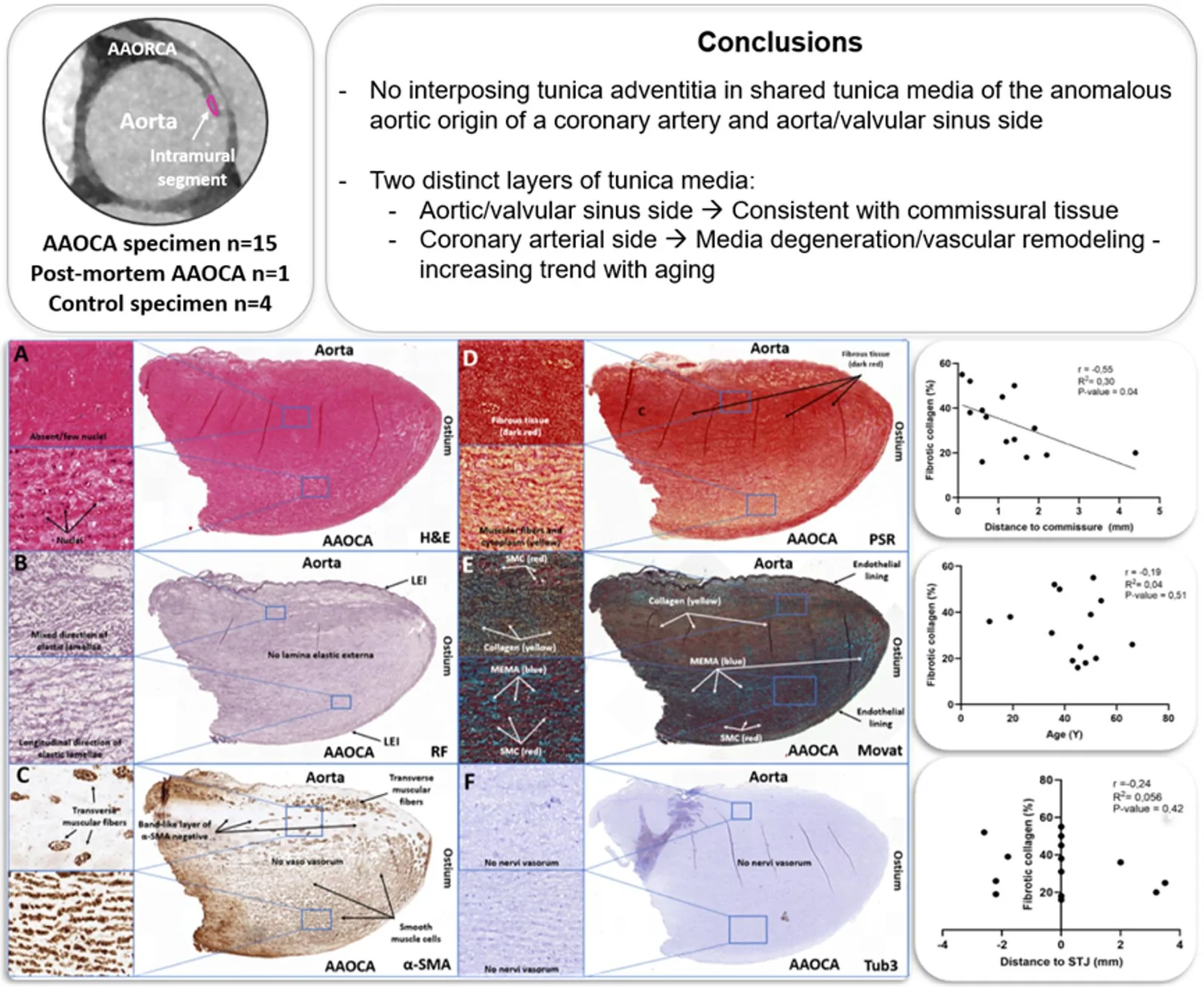

**Supplementary Information:**

The online version contains supplementary material available at https://doi.org/10.1186/s12872-026-06000-7.

## Introduction

Anomalous aortic origin of a coronary artery (AAOCA), a congenital heart disease in which both coronary arteries arise from the same aortic sinus, occurs in 0.1%-1% of the general population [1–3]. AAOCA is associated with an increased risk for acute myocardial ischemia and sudden cardiac death (SCD), especially in the presence of high-risk anatomical features, such as a slitlike orifice, an acute-angle take-off, and an interarterial intramural course [4]. The European Society of Cardiology and American Heart Association guidelines advise to pro-actively identify these high-risk anatomical features in AAOCA patients [5, 6]. In living patients, computed tomography angiography (CTA) is routinely used to stratify the anatomy. An unambiguous diagnosis of an intramural course is challenging without available histology. In literature, an intramural course is described as “a segment in which the AAOCA and aorta share the tunica media”, as determined through post-mortem studies [7–11]. The shared vascular wall segment is also referred to as the “intramural segment“, and by definition has a coronary arterial side and an aortic side. In living patients, the intramural segment can be evaluated using CTA [8]. Knowledge of the histological characteristics of the intramural segment from living AAOCA patients is scarce.

Theoretically, structural vascular abnormalities may influence the compliance of the intramural segment, leading to increased or decreased rigidity of the AAOCA vascular wall. This may influence its collapsibility under external compression or predispose to the formation of a valve-like ridge at the AAOCA ostium. Such a valve-like ridge is believed to develop from the proximal part of the intramural segment. A more compliant segment would be more prone to act as a flap valve impeding coronary arterial flow through the coronary ostium during exercise or under high aortic pressure, potentially resulting in SCD [12–14]. Data on this are, however, scarce.

The aim of this study was to identify the histological features of the intramural segment and their potential relevance with respect to vascular wall properties and the aortic valve commissure, thereby enhancing understanding of its role in pathophysiology and clinical outcomes of patients with AAOCA.

## Methods

This multicenter prospective cohort study was conducted in three academic referral centers for Adults with Congenital Heart Disease (ACHD), i.e. the Leiden University Medical Center (LUMC), the Amsterdam University Medical Center (Amsterdam UMC) and the University Medical Center Groningen (UMCG). All consecutive pediatric and adult patients who underwent surgical unroofing of an AAOCA between January 2021 and January 2024 were eligible for inclusion. Patients with concomitant congenital heart disease were excluded. Demographic and clinical data were retrieved from the electronic health record systems (EPD-Vision©, Leiden University Medical Center, Leiden, the Netherlands, EPIC, Epic Systems, Verona, Wisconsin, USA, and HiX, Chipsoft, Amsterdam, the Netherlands). CTA measurements were performed in a blinded fashion by an experienced investigator, DBHV, as previously described [15]. Coronary anatomy was defined according to the Leiden Convention coronary coding system [16]. The intramural segment was assessed by measuring the intraluminal space (ILS), a parameter of the intramural AAOCA segment, as previously described [8]. Indication for the unroofing procedure was decided on by a multidisciplinary team including Adult Congenital Heart Disease (ACHD) cardiologists, interventional cardiologists and cardio-thoracic surgeons specialized in ACHD.

### Unroofing procedure, tissue collection and preservation

The unroofing procedure is illustrated in Figure S1 and Video S1. The intramural segment was fully unroofed and was marked with a surgical suture or sterile marker for orientation purposes before 24–48 h fixation in 4% paraformaldehyde (PFA). The tissue was stored in 70% ethanol.

### Postmortem tissue

To enhance understanding of the spatial correlation of the intramural segment and its histology, a postmortem heart specimen with AAOCA with an intramural course from the Leiden Collection of Congenital Malformations was analyzed. This specimen is part of the Biobank Congenital Heart Disease of the Leiden University Medical Center (B21.051). Use of the postmortem heart for the current study was approved by the LUMC Review Committee Biobank & biomaterials (TCBio) under protocol number RP24.001. Post-mortem magnetic resonance imaging (MRI) of this specimen was performed. Furthermore, a complete cross-section of the aorta and AAOCA was obtained, enabling histological assessment of the intramural segment within the surrounding aortic and AAOCA tissue.

### Control tissue of subjects with normal coronary anatomy

For comparison of the outcomes from the histological assessment of the aortic and coronary arterial tissue from AAOCA patients, tissue from subjects with normal coronary anatomy, all with a coronary artery origin from the aortic sinus and normal course, were included. These aortic and coronary arterial tissue samples were obtained from deceased patients without AAOCA who have given written consent for body donation for teaching and scientific research purposes. Tissue was embedded according to Fix for Life Embalming, isolated, and stored in 70% ethanol until further procedures were conducted [17].

### Histological and immunohistochemical staining and histological analysis

All unroofing, post-mortem, and control tissue samples were embedded in paraffin. Microtomic sections of 5 μm were serially mounted on glass slides (Klinipath). Standard protocol for histological staining Hematoxylin & Eosin (H&E), resorcin fuchsin (RF), Movats pentachrome (Movat) and Sirius Red (SR) were followed. For immunohistochemical staining, sections were incubated with primary antibodies, followed by incubation with a peroxidase-coupled secondary antibody, and hematoxylin counterstain, Table 1. All sections were mounted with Entellan until high-resolution photomicrographs for each section were captured using a 3DHistech Panoramic 250 Flash III digital scanner at 20× magnification.

**Table 1:** Histological stains and assessments

Histochemical and immunohistochemical staining	Analysis parameters per staining
Hematoxylin-eosin stain (H&E)	- Presence of nuclear components - Presence of cytoplasmic components including collagen and elastic fibers, muscle fibers, and red blood cells
Movat pentachrome (Movat)	- Morphology and organization of elastic fibers (thinning, fragmentation/loss) - Morphology and organization of smooth muscle cells - Collagen alterations and fibrous tissue - Mucoid Extracellular Matrix Accumulation (MEMA)
Resorcin fuchsin (RF)	- Morphology and organization of elastic fibers (thinning, fragmentation/loss) - Intima thickening
Sirius Red (SR)	- Collagen alterations and fibrous tissue
α smooth muscle actin (α-SMA, A2547, Sigma-Aldrich, 1:10.000)	- Morphology and organization of smooth muscle cells
Platelet endothelial cell adhesion molecule-1 (PECAM-1, sc-1506-R, Santa Cruz, 1:500)	- Gap junctions and adherence junctions (endothelial lining) - Vaso-vasorum
βIII-tubulin (Tub3, T2200-200UL, Sigma Aldrich, 1:500)	- Subepicardial and myocardial autonomic nerves (nervi vasorum)
Goat anti-rabbit secondary (BA-1000, Vector Laboratories, 1:250)	
Horse anti-mouse secondary (BA-2000, Vector Laboratories, 1:250)	

### Histopathology and qualitative data analysis

For all included tissue specimens, a scoring form, based on the consensus statement on surgical pathology of the aorta from the Society for Cardiovascular Pathology and the Association for European Cardiovascular Pathology [18], was systematically completed by two independent observers (DBHV & TCB). Any discrepancies were resolved through reassessment until consensus was achieved. The scoring form is available in the supplemental material, Text S1.

### Quantification of fibrous tissue and mucoid extracellular matrix accumulation

The automated pixel classification of QuPath image analysis software was used for quantification of the fibrous tissue and mucoid extracellular matrix accumulation (MEMA) [19]. Slides were loaded into the program, and via script, all stats and staining vectors were set identically. Fibrous tissue was quantified via SR staining and α-Smooth Muscle Actin, clone 1A4 (α-SMA) staining. MEMA was quantified via Movat staining. After annotations were set, the pixel classifier ran, and the output was exported.

### Current literature

To discuss our findings in light of current literature on histological characteristics of intramural AAOCA and expose potential gaps in knowledge, a comprehensive literature search was conducted in the PubMed, PMC PubMed Central, Embase, Web of Science, Cochrane Library, Emcare, Academic Search Premier, ScienceDirect, and Google Scholar databases. The search strategies included “Anomalous aortic origin of a coronary artery”, “Intramural course” and “Tunica Media”. Full queries are detailed in Supplemental Text S2. Full text articles, including intramural AAOCA, as well as a histological assessment, were evaluated and discussed in light of current findings in the Discussion section of the manuscript where appropriate.

### Statistical analysis

Statistical analysis was performed using SPSS (version 25; SPSS Inc, Chicago, USA) and GraphPad Prism (version 10.2.3 for Windows, GraphPad Software, Boston, Massachusetts USA). Normally distributed continuous data were presented as mean ± standard deviation (SD), and not normally distributed continuous data as median and the first to third interquartile range [IQR1-IQR3]. Categorical data were reported as numbers and percentages. The relation between continuous data were assessed with the Pearson Correlation and differences in means with the Unpaired T-test and Mann–Whitney U test. A *p*-value of < 0.05 was considered statistically significant.

## Results

### Patient characteristics

Fifteen consecutive patients (mean age 42.9 ± 14.0 years, age range 11–66 years, 9 (60%) female) were prospectively included, Table 2 and Figure S2. Fourteen (93%) patients had an anomalous aortic origin of a right coronary artery (AAORCA) and 1 (7%) patient had an anomalous aortic origin of a left coronary artery (AAOLCA). All AAOCA had an interarterial course. Two (13%) patients had a history of hypertension, and one (7%) patient had aortic dilatation up to 50 mm with severe aortic valve regurgitation. This patient underwent concomitant aortic valve and aneurysm surgery (Bentall procedure).

**Table 2:** Patient characteristics

	Patients *n* = 15
Female, *n* (%)	9 (60)
Age first diagnosis AAOCA (years), mean ± SD	42.9 ± 14.0
BMI, mean ± SD	25.8 ± 5.0
Anatomy according to the Leiden convention coronary coding system, n (%)	
* 1R*, LCx*	1 (7)
2R*,*LCx*	13 (87)
2R*,*L*,* Cx*	1 (7)
History, n (%)	
* Hypertension*	2 (13)
* Pulmonary arterial hypertension*	0
* Myocardial infarction*	0
* Supraventricular arrhythmia*	2 (13)
* Ventricular arrhythmia*	2 (13)
* Premature ventricular complex*	2 (13)
* Non-sustained ventricular tachycardia*	1 (7)
Aortic dilatation with aortic valve regurgitation	1 (7)
Symptoms at presentation, n (%)	
* Typical angina*	6 (40)
* Atypical chest pain*	4 (27)
* Non-anginal chest pain*	2 (13)
* Dyspnea*	4 (27)
* Palpitations*	3 (20)
* (Pre-)Syncope*	1 (7)
* Incidental finding*	0
Result ischemia testing in the matching territory, n (%)	
* Positive*	6 (40)
* Negative*	8 (53)
* Not performed*	1 (7)
Primary indication for surgery, n (%)	
* AAOCA*	14 (93)
* Aortic dilatation with severe aortic valve regurgitation + AAOCA*	1 (7)

*Abbreviations*: *AAOCA* anomalous aortic origin of a coronary artery, *ACEI* Angiotensin-Converting Enzyme Inhibitors, *ARB* Angiotensin II Receptor Blockers, *BMI* Body mass index, *CCB* Calcium channel blockers

### Computed tomography angiography

CTA showed an interarterial course in all patients, Table 3. The right coronary artery was dominant in all patients with AAORCA except one. In the patient with AAOLCA, right coronary dominance was preserved. A slitlike orifice shape was observed in 12 (80%), an acute coronary artery angle take-off in 14 (93%), and proximal narrowing in 14 (93%) patients. Twelve patients (80%) had a coronary ostium originating from the aortic sinus at or below the sinutubular junction (STJ), with seven ostia located at the level of the STJ and five below it. In contrast, three patients (20%) had a coronary ostium originating from the tubular aorta above the STJ. Median distance from the coronary ostium to the aortic valve commissure was 1.1 mm [IQR 0.4–1.9 mm].

**Table 3:** Anatomical features on CTA before the unroofing procedure

	Patients *n* = 15
Anatomy according to Leiden convention, *n* (%)	
* 1R*, LCx*	1 (7)
2R*,*LCx*	13 (87)
2R*,*L*,* Cx*	1 (7)
Interarterial course, n (%)	15 (100)
Intramural course (ILS < 0.95 mm at 2 mm from ostium), n (%)	15 (100)
Measurements ILS, mean ± SD	
* ILS < 0.95 mm at 2 mm from ostium*	0.7 ± 0.4
* Distance ostium to ILS > 0.95 mm*	
* AAORCA*	7.8 ± 3.6
* AAOLCA*	16.0
Cardiac dominance, n (%)	
AAOCA	
* AAORCA*	13 (87)
* AAOLCA*	0
Non-AAOCA	2 (13)
Take-off in relation to STJ, n (%)	
* Above*	3 (20)
* At the level of*	7 (47)
* Below*	5 (34)
Distance to STJ in mm, median [IQR]	0 [-1.9-0.5]
Distance to commissure in mm, median [IQR]	1.1 [0.4–1.9]
Orifice shape, n (%)	
* Round*	0
* Oval*	3 (20)
* Slitlike*	12 (80)
Coronary take-off angle, mean ± SD	21.8 ± 6.0
Acute coronary take-off angle (< 30°), n (%)	14 (93)
Proximal narrowing, n (%)	14 (93)

*Abbreviations*: *AAOCA* anomalous aortic origin of a coronary artery, *AAOLCA* anomalous aortic origin of a left coronary artery, *AAORCA* anomalous aortic origin of a right coronary artery, *CTA* computed tomography angiography, *ILS* interluminal space, *STJ* sinutubular junction

### Histological characteristics of control specimens

**Fig. 1:**
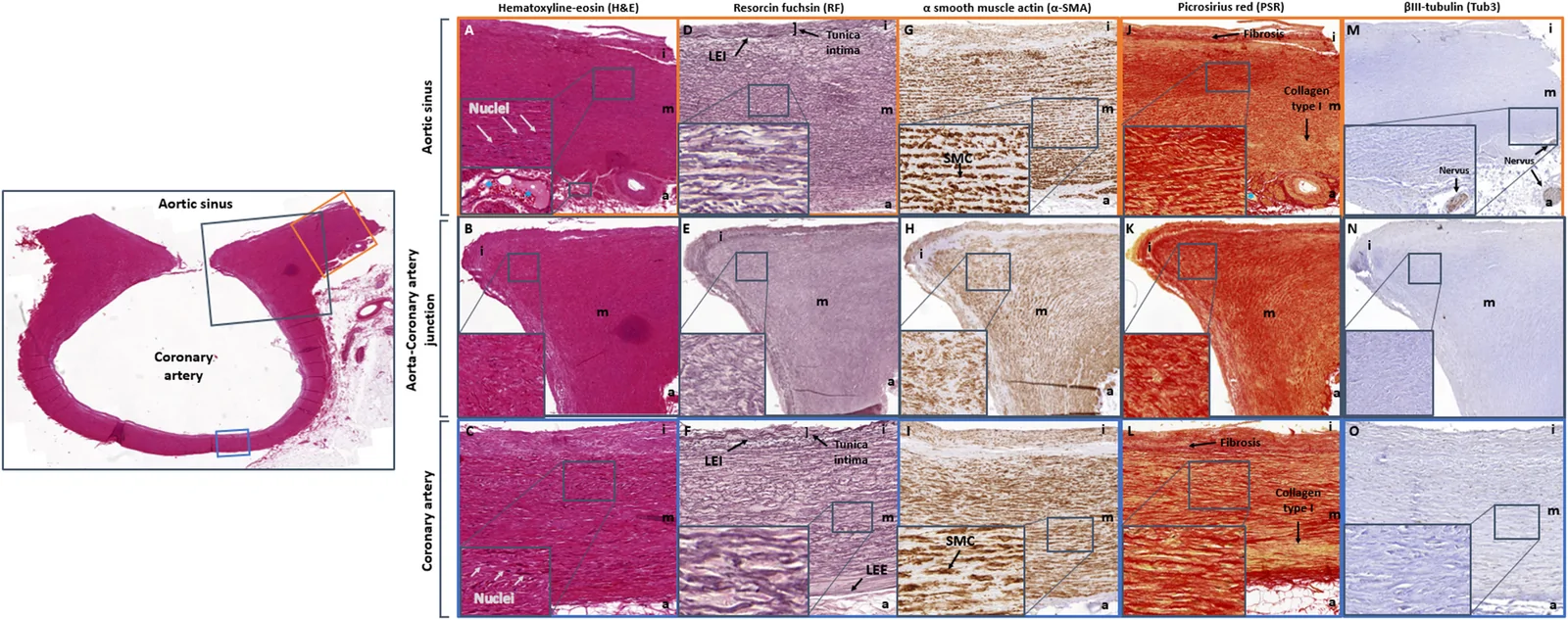
Histological characteristics of the aortic sinus and coronary artery vascular wall of a patient with normal coronary anatomy; 1R, 2LCx according to The Leiden Convention coronary coding system. The central left panel provides an overview of the histological section, indicating the locations of the higher-magnification images shown in the right panels. The upper right panels illustrate the aortic sinus vascular wall, the middle right panels the aortic-coronary artery junction vascular wall and the lower right panels the coronary arterial vascular wall. Abbreviations: *, red blood cells; a, tunica adventitia; i, tunica intima; LEE, lamina elastica externa LEI, lamina elastica interna ;m, tunica media; SMC, smooth muscle cell

The histological characteristics of the vascular wall of the aortic sinus, aorta-coronary artery junction, and coronary artery of 4 patients with normal coronary anatomy are displayed in Table 4 and Fig. 1A-O. In all control samples, the tunica intima was characterized by a lamina elastica interna and endothelial lining, Fig. 1D-F. In one specimen intima thickening was observed in the tunica intima of the coronary artery. The tunica media of the aortic sinus, aorta-coronary artery junction, and coronary artery were characterized by the presence of nuclei, smooth muscle cells, and elastic lamellae, Fig. 1A-I. The orientation of the elastic lamellae within the aortic sinus vascular wall was longitudinal in 3 (75%) and multidirectional, showing both intermingled longitudinal and circumferential directions, in 1 (25%), whereas in the aorta-coronary artery junction and coronary arterial vascular wall, the multidirectional pattern was more frequently observed, Fig. 1D-F. The mean thickness of the elastic lamellae in the aortic sinus, aorta-coronary artery junction, and coronary artery were 2.6 ± 0.8 μm, 2.0 ± 0.7 μm, and 1.8 ± 0.6 μm, respectively. In three controls, a lamina elastica externa was identified within the tunica media of the coronary artery (and not the aortic sinus), and in 1 control, no lamina elastica externa was identified, Fig. 1D-F.

**Table 4:** Histological characteristics of the vascular wall of the aortic sinus, aorta-coronary junction, and coronary artery of control specimens with regular coronary anatomy; 1R,2LCx according to The Leiden Convention coronary coding system, of the vascular wall of the aortic side and coronary arterial side of the AAOCA specimen, and of the vascular wall of the aortic lumen side and aortic outer border side of an aortic valve commissure which is not opposite to the anomalous coronary artery of the aorta in the post-mortem specimen

	Control specimens			AAOCA specimens intramural segment		Post-mortem specimen aortic valve commissure	
		
	Aorta *n* = 4	Aorta-coronary artery junction *n* = 4	Coronary artery *n* = 4	Aortic side *n* = 15	Coronary arterial side *n* = 15	Aortic lumen side	Aortic outer border side
Tunica intima							
Intima thickening (width > 0,5x mid segment), n (%)	0	0	1 (25)	0	1 (8)	0	NA
Lamina elastica interna visible, n (%)	4 (100)	4 (100)	4 (100)	12 (80)	12 (92)	1 (100)	NA
Endothelial lining present, n (%)	4 (100)	4 (100)	4 (100)	10 (67)	11 (85)	1 (100)	NA
Tunica media							
Presence of smooth muscle cells, n (%)	4 (100)	4 (100)	4 (100)	13 (87)	15 (100)	1 (100)	1 (100)
Band-like layer without smooth muscle cells, n (%)	0	0	0	14 (93)	4 (27)	1 (100)	0
Presence of elastic lamellae, n (%)	4 (100)	4 (100)	4 (100)	15 (100)	15 (100)	1 (100)	1 (100)
Direction of elastic lamella, n (%)							
* Longitudinal*	3 (75)	1 (25)	1 (25)	3 (20)	9 (60)	0	1 (100)
* Circular*	0	0	0	3 (20)	1 (7)	0	0
* Multidirectional*	1 (25)	3 (75)	3 (75)	9 (60)	5 (33)	1 (100)	0
Thickness of elastic lamella (µm), mean ± SD	2.6 ± 0.8	2.0 ± 0.7	1.8 ± 0.6	2.2 ± 0.4	2.3 ± 0.4	1.9 ± 0.2	2.1 ± 0.3
Presence of lamina elastica externa, n (%)	0	0	3 (75)	0	0	0	0
Presence of nuclei, n (%)	3 (100)*	3 (100)*	3 (100)*	15 (100)	15 (100)	1 (100)	1 (100)
Areas with fibrosis/fibrous tissue, n (%)	3 (75)	4 (100)	3 (75)	15 (100)	14 (93)	1 (100)	1 (100)
Transverse sections of muscle fiber bundles present, n (%)	0	2 (50)	0	10 (67)	0	1 (100)	0
Vasa vasorum, n (%)	0	0	0	0	0	0	0
Nervi vasorum, n (%)	0	0	0	0	0	0	0
Tunica adventitia							
Vasa vasorum, n (%)	4 (100)	4 (100)	4 (100)	NA	NA	NA	1 (100)
Nervi vasorum, n (%)	4 (100)	4 (100)	4 (100)	NA	NA	NA	1 (100)

*In 1 control the presence of nuclei could not be determined due to inconclusive staining of Hematoxylin-eosin stain (H&E) stain

Limited areas of fibrous tissue (defined as alpha smooth muscle negative, Sirius Red positive) within the tunica media were observed in all control samples; areas of fibrous tissue in the aortic sinus in 3 (75%), in the aorta-coronary artery junction in 4 (100%), and in the coronary artery in 3 (75%), Fig. 1J-L. Transverse smooth muscle fibers were identified in the media in 2 (50%) of the controls. No vasa vasorum or nervi vasorum were observed within the tunica media of any controls, however, these structures were present in the tunica adventitia of all samples, Fig. 1M-O.

### Histological characteristics of the AAOCA specimens

**Fig. 2:**
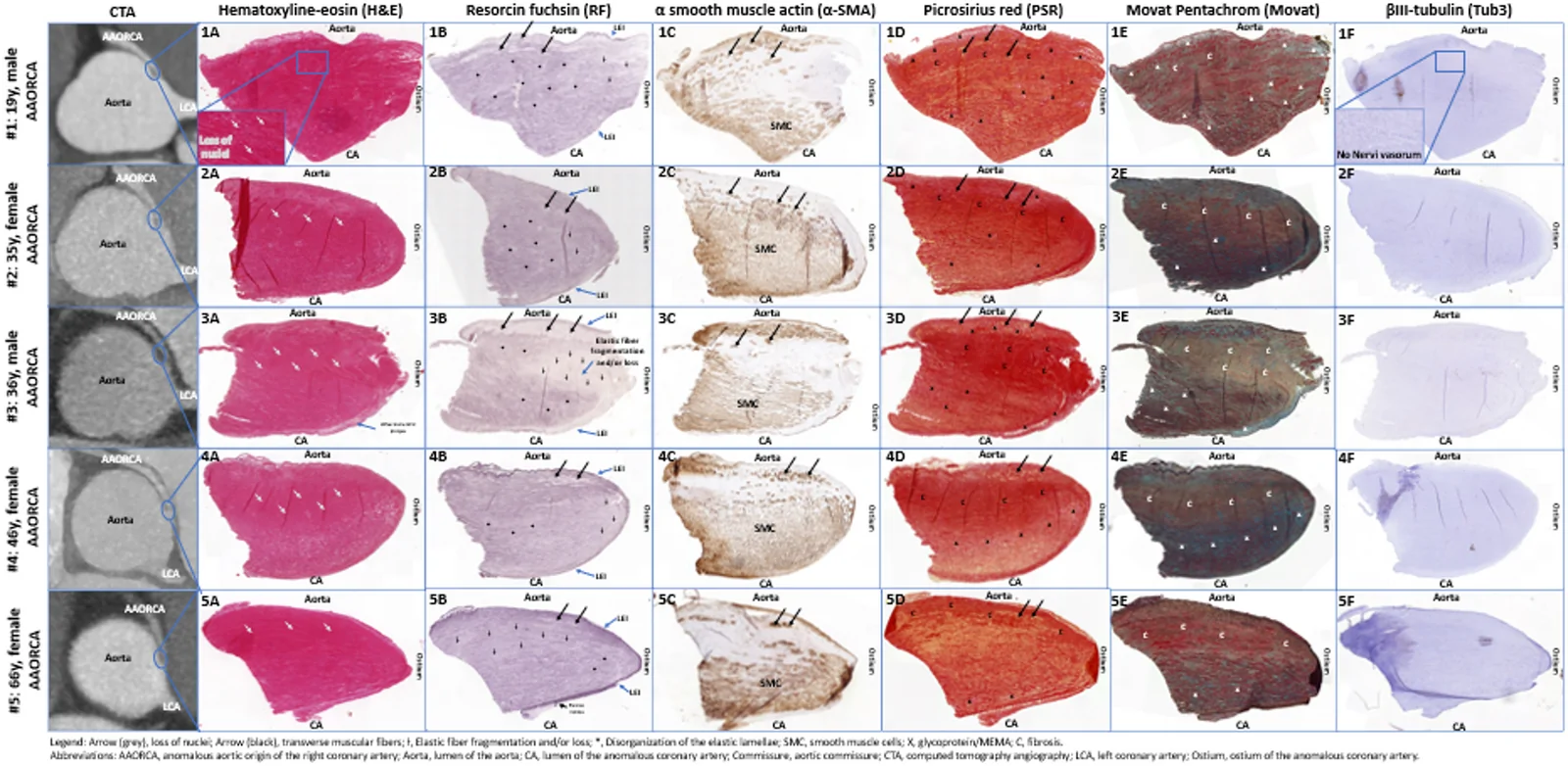
Overview of the corresponding CTA images (first column) and observed histological patterns of the intramural segment (second to seventh column) of 5 included patient. Legend: Arrow (grey), loss of nuclei; Arrow (black), transverse muscular fibers; ƚ, Elastic fiber fragmentation and/or loss; *, Disorganization of the elastic lamellae; SMC -, area without smooth muscle cells; X, glycoprotein/MEMA; C, fibrous tissue of aortic valve commissure. Abbreviations: AAORCA, anomalous aortic origin of the right coronary artery; Aorta, lumen of the aorta; CA, lumen of the anomalous coronary artery; Commissure, aortic valve commissure; CTA, computed tomography angiography; LCA, left coronary artery; Ostium, ostium of the anomalous coronary artery

**Fig. 3:**
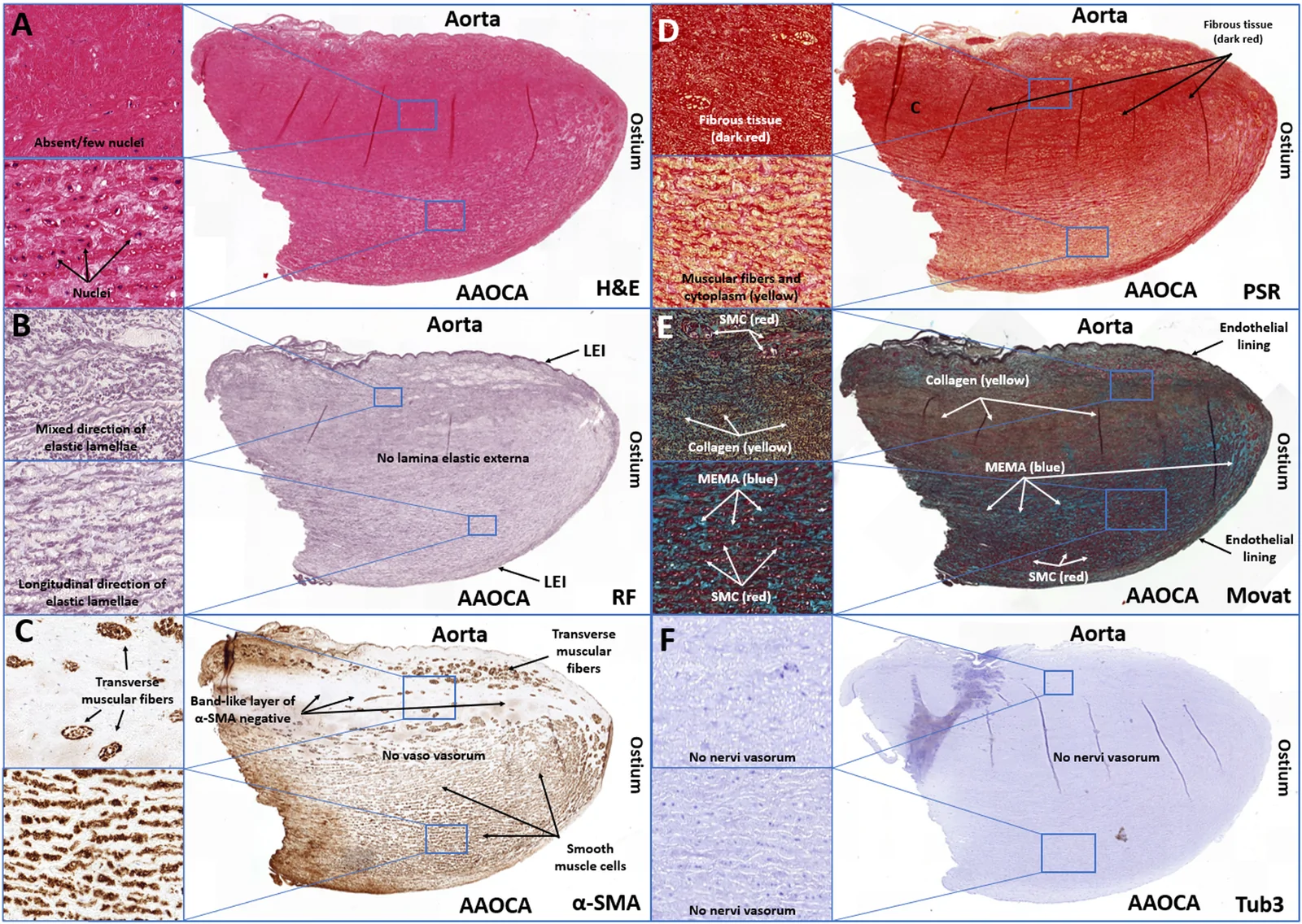
Histological characteristics of the intramural segment in patient #4 shown in Fig. 2. Panels **A**–**F** represent different histological stainings, as indicated in the lower right corner of each panel. Within each panel, the central image provides an overview of the section, whereas the upper and lower left images show higher-magnification views of the observed histological features. Abbreviations: AAOCA, anomalous aortic origin of the coronary artery; LEI, lamina elastica interna; MEMA, mucoid extracellular matrix accumulation; SMC, smooth muscle cells

A total of 15 specimens were included and analyzed, Table 4, Figs. 2 and 3. In 13 (86%) samples, both the aortic and the coronary arterial sides could be identified.

#### Tunica intima

In 2 (13%) samples, the tunica intima on the coronary arterial side could not be identified. Endothelial lining was identified in 10/15 (67%) on the aortic side (sinus or tubular aorta, depending on the AAOCA origin) and in 11/13 (85%) on the coronary arterial side. A lamina elastica interna was observed in 12/15 (80%) on the aortic side and in 12/13 (92%) on the coronary arterial side, Fig. 3B. In one (8%) patient, intima thickening of the coronary artery was observed.

#### Tunica media

In all sections, two ill-defined layers with different histological characteristics were identified in the area medial from the tunica intima on the aortic sinus or tubular aortic side and the coronary arterial side, based on the expression pattern of alpha smooth muscle actin, Sirius Red (SR), and Movat, Fig. 3C-E. In none of the specimens were characteristics of a tunica adventitia, such as a vasa vasorum or nervi vasorum, observed in this layer in the intramural segment, Fig. 3C, F. Therefore, this was considered a two-layered shared tunica media without interposing tunica adventitia. Differentiating characteristics between the tunica media of the aortic sinus/tubular aortic side and the coronary arterial side were: a band-like layer without smooth muscle cells (α-SMA negative) and more pronounced fibrous tissue (α-SMA negative and SR positive) in the tunica media on the aortic side in 13 (87%) vs. 4 (27%) on the coronary arterial side, Fig. 3C-D; elastic lamellae with a structured longitudinal direction in 3 (20%) on the aortic side vs. 9 (60%) on the coronary arterial side, Fig. 3B; multidirectional disorganized directions of the elastic lamellae in 9 (60%) on the aortic side vs. 5 (33%) on the coronary arterial side, Fig. 3B, and the presence of transverse sections of muscle fiber bundles in 10 (67%) on the aortic side vs. none on the coronary arterial side, Fig. 3C.

Although several media characteristics were similar, the band-like layer, which lacked smooth muscle cells (α-SMA negative) and contained high amounts of fibrous tissue (SR positive) in the intramural segments of the AAOCA, was not observed in control specimens.

#### Tunica adventitia

In the AAOCA specimens, no characteristics of a tunica adventitia, such as vasa vasorum and nervi vasorum, were observed, Fig. 3C, F.

### Histological characteristics of the post-mortem AAOCA specimen

**Fig. 4:**
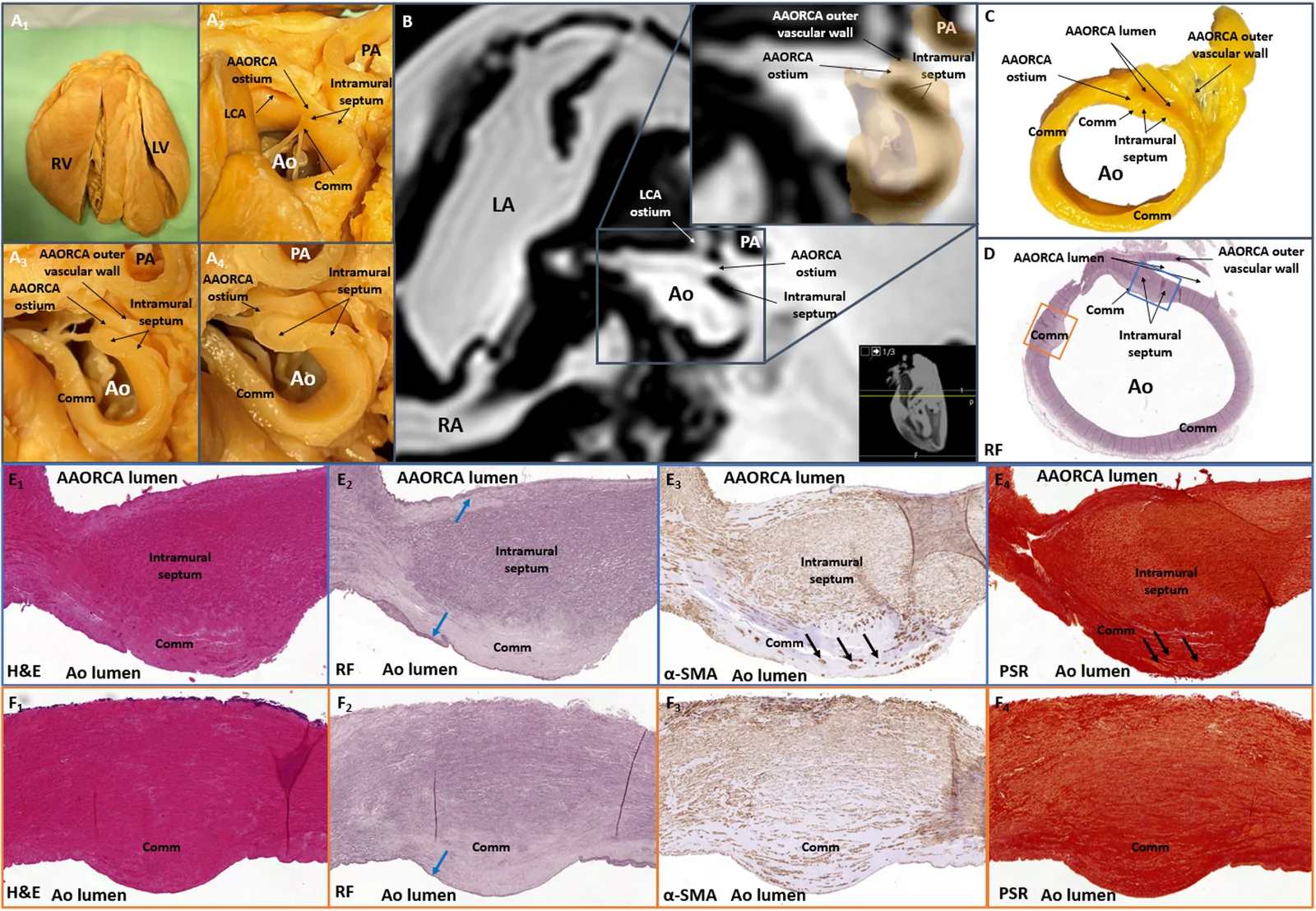
Macroscopic imaging of the post-mortem specimen with an AAORCA in which the aorta is dissected perpendicular at the level of the origin of the AAORCA (**A**_**1−4**_); Frontal view (**A**_**1**_), lateral view (**A**_**2**_), and overhead view (**A**_**3**_), and cranial view (**A**_**4**_). Panel **B** shows the post-mortem MRI image of the heart at the level of the aortic sinus. A macroscopic image of the superior segment of the aortic sinus including the origin of the AAORCA and the superior part of the proximal AAORCA vessel with the intramural segment (**C**). Panel **D** shows the histological characteristics of the complete transverse section of the aortic sinus at the level of the aortic valve commissures and AAORCA ostium at the upper side of the section with the intramural segment and proximal part of the outer vascular wall of the AAORCA with RF staining. Panels **E1-4** show the intramural segment (blue box in panel **D**) and panels **F**_**1−4**_ show the noncoronary-left coronary commissure (orange box in panel **D**) with H&E, RF, α-SMA and SR staining. Abbreviations: AAORCA, anomalous aortic origin of a right coronary artery; Ao, aorta; Comm, commissure; Arrow (black), transverse muscular fibers; Arrow (blue), Lamina elastica interna; H&E, Hematoxylin-eosin; LCA, left coronary artery; LV, left ventricle; PA, pulmonary artery; SR, Sirius Red; RF, Resorcin fuchsin; RV, right ventricle; α-SMA, α smooth muscle actin

One post-mortem heart with an interarterial AAORCA (2R*,LCx according to the Leiden convention coronary coding system), from a patient who suffered sudden death due to an unknown cause, was available for analysis, Table 4 and Fig. 4. In contrast to the surgical sections, in this specimen, the entire circumference of the aortic wall, including part containing the intramural segment, was available for analysis. Macroscopic inspection and post-mortem MRI imaging enhanced the understanding of the spatial relation of the AAORCA ostium and intramural segment, by which a close spatial anatomical relationship with the aortic valve commissure was noted, Fig. 4A-C.

#### Tunica intima

Histological assessment revealed a similar pattern of the tunica intima of the intramural segment as the surgical sections, with endothelial lining and a lamina elastica interna on both the aortic sinus and coronary arterial side of the intramural segment, Fig. 4E_2_.

#### Tunica media

Similar to the prospectively included AAOCA specimens, two ill-defined layers with different histological characteristics were identified in the tunica media. The tunica media of the intramural segment was characterized by multidirectional disorganized directions of the elastic lamellae, Fig. 4E_2_, absence of a lamina elastica externa, Fig. 4E_2_, presence of transverse sections of muscle fiber bundles, Fig. 4E_3_, the same band-like layer on the aortic sinus side with severe reduction of smooth muscle cells, Fig. 4E_3_, and a comparable collagen pattern with dense expression of SR, Fig. 4E_4_, similar to the prospectively included AAOCA specimen.

Histological assessment of the aortic vascular wall at the location of the valve commissures (i.e. noncoronary-left coronary commissure and the noncoronary-right coronary commissure), which had no relation with the anomalous coronary arterial ostium, revealed a similar histological pattern, e.g. a band-like layer without smooth muscle cells (α-SMA negative) and more pronounced fibrous tissue (α-SMA negative and SR positive), in the (non-shared) aortic sinus wall as observed in the intramural segment of the AAOCA specimens, Fig. 4F_1−4_. These vascular wall characteristics were not observed in the aortic sinus wall between the valve commissures. Therefore, it was considered that the observed vascular wall characteristics on the aortic side of the intramural segment in AAOCA represent aortic valve commissure.

#### Tunica adventitia

**Fig. 5:**
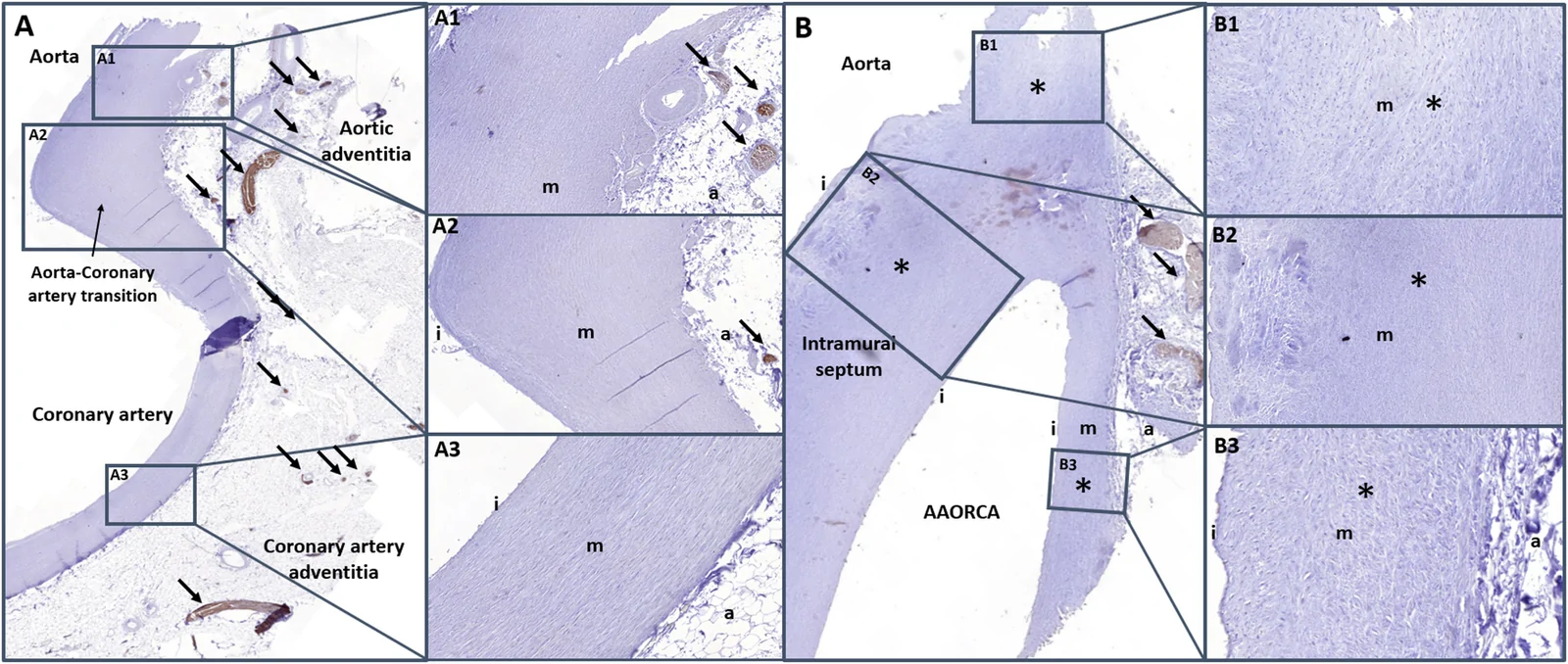
βIII-tubulin (Tub3) staining of the aorta, coronary artery and aorta-coronary artery junction of a specimen with normal coronary anatomy (**A**) and a specimen with an anomalous aortic origin of a right coronary artery (**B**) revealing nervi vasorum in the tunica adventitia (black arrows in A and B) in both specimens. No nervi vasorum was observed in the shared medial wall the AAOCA specimen (asterisk in B). Abbreviations: a, tunica adventitia; AAORCA, anomalous aortic origin of a right coronary artery; i, tunica intima; m, tunica media

Similar to the surgical sections, no vasa and nervi vasorum were present in the intramural segment of the shared aortic sinus and coronary arterial vascular wall, and no tunica adventitia was observed. In the remainder of the aortic wall of the post-mortem AAOCA specimen, excluding the intramural segment, a tunica adventitia with nervi and vasa vasorum was present, Fig. 5.

### Spatial relation between the AAOCA ostium and aortic valve commissure

**Fig. 6:**
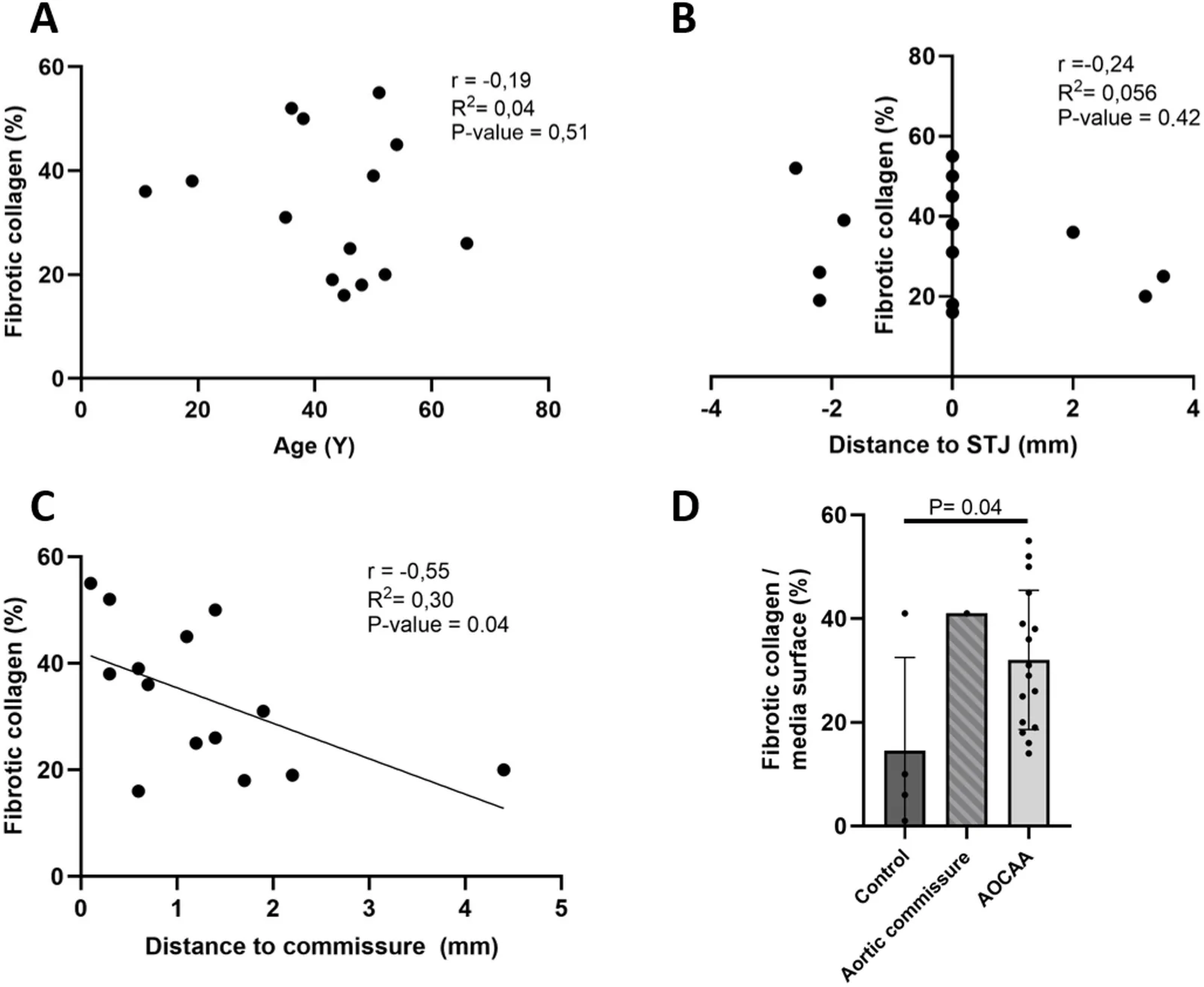
Scatterplots illustrating the relation between the percentage of fibrotic collagen and age (**A**), the relation between the percentage of fibrotic collagen and distance to the sinutubular junction (**B**), and the relation between the percentage of fibrotic collagen and distance of the AAOCA ostium to the aortic valve commissure (**C**). Panel **D** demonstrates the bar graph illustrating the percentage of fibrous tissue in the control specimens, a specimen of aortic valve commissure not related to a coronary artery in the post-mortem specimen, and the AAOCA specimens. Abbreviations: AAOCA, anomalous aortic origin of a coronary artery; STJ, sinutubular junction

To further substantiate the presence of aortic valve commissure at the aortic side (aortic sinus/tubular aorta) of the intramural segment, the distance between the AAOCA ostium and aortic valve commissure was measured on the pre-operative CTA. For this analysis, 1 (7%) patient was excluded due to a concomitant aortic aneurysm. The median distance from the AAOCA ostium to the valve commissure was measured and found to be 1.1 mm [IQR 0.4–1.9 mm] and the median distance from the AAOCA ostium to the sinutubular junction was 0 [IQR 1.9 mm under the sinutubular junction (STJ) to 0.5 mm above STJ], Table 3. Quantitative assessment of the amount of fibrous tissue in the AAOCA specimens demonstrated a mean area of 29.1% (SD ± 15.5%) of the band-like layer without smooth muscle cells (α-SMA negative) and with fibrous tissue (SR positive) in the intramural segments of the AAOCA. No significant relation was observed between the age of the patients and the percentage of the fibrous tissue, (*p* = 0.51), Fig. 6A. Furthermore, no significant relationship was observed between the distance of the AAOCA ostium to the sinutubular junction and the percentage of fibrous tissue (*p* = 0.42), Fig. 6B. However, a weak yet significant inverse relation was observed between the distance of the AAOCA ostium to the aortic valve commissure and the amount of fibrous tissue, with decreasing amount of fibrous tissue as distance to the aortic valve commissure increased (*R*=-0.55, R^2^ = 0.3, *p* = 0.04), Fig. 6C. Furthermore, the fibrotic collagen ratio to surface area of the tunica media of the AAOCA specimen was similar to that of the aortic valve commissure and significantly different compared to the ratio in the tunica media of the controls (*p* = 0.04), Fig. 6D. This substantiated the interpretation that the vascular wall characteristics on the aortic sinus and tubular aortic side reflect the aortic valve commissure.

### Vascular wall characteristics at the coronary arterial side of the intramural segment

The observed vascular characteristics of the tunica media on the coronary arterial side of the intramural segment were unlikely to be entirely attributed to aortic valve commissural tissue. In contrast to the aortic side of the intramural segment, which was characterized by dense (alpha smooth muscle cell negative and Sirius red positive) fibrous tissue, the coronary arterial side of the intramural segment was characterized by increased glycoprotein accumulation as appreciated in the Movat staining, Fig. 3D-E. Furthermore, the vascular characteristics compatible with aortic valve commissural tissue were confined to the tissue bordering the aortic lumen of the vascular wall at the location of the commissures (i.e. noncoronary-left coronary commissure and the noncoronary-right coronary commissure).

The tunica media on the coronary arterial artery side of the intramural segment and control specimens were quantitatively graded, Table 5 and Table S1. Features of media degeneration, such as mucoid extracellular matrix accumulation (MEMA), elastic fiber fragmentation and/or loss, elastic fiber thinning, smooth muscle cell nuclei loss, and collagen alterations, all appeared more frequently and/or were more severe in the tunica media of the AAOCA specimens compared to the control specimens. This tendency was supported by the overall media degeneration score, which was more severe in the AAOCA specimens compared to the controls.

**Table 5:** Outcomes of quantitative grading of the tunica media of the AAOCA specimens

	Control specimens			
	Aorta *n* = 4	Aorta-coronary artery junction *n* = 4	Coronary artery *n* = 4	Coronary arterial side *n* = 15
Overall media degeneration, *n* (%)				
* Mild*	4 (100)	0	2 (50)	2 (13)
* Moderate*	0	3 (75)	1 (25)	12 (80)
* Severe*	0	1 (25)	1 (25)	1 (7)
MEMA, n (%)				
* Mild*	-	-	-	1 (7)
* Moderate*	-	-	-	11 (79)
* Severe*	-	-	-	2 (14)
* Inconclusive*	4*	4*	4*	-
Elastic fiber fragmentation and/or loss, n (%)				
* Absent*	0	0	0	1 (7)
* Mild*	4 (100)	3 (75)	1 (25)	3 (20)
* Moderate*	0	1 (25)	1 (25)	6 (40)
* Severe*	0	0	2 (50)	5 (33)
Elastic fiber thinning, n (%)				
* Absent*	1 (25)	0	0	0
* Mild*	3 (75)	0	1 (25)	4 (27)
* Moderate*	0	2 (50)	1 (25)	3 (20)
* Severe*	0	2 (50)	2 (50)	8 (53)
SMC nuclei loss, n (%)				
* Mild*	2 (67)	0	1 (33)	10 (67)
* Moderate*	0	2 (67)	1 (33)	3 (20)
* Severe*	1 (33)	1 (33)	1 (33)	2 (13)
* Inconclusive*	1*	1*	1*	-
Laminar medial collapse, n (%)				
* Absent*	1 (25)	0	0	0
* Mild*	1 (25)	0	2 (50)	8 (53)
* Moderate*	2 (50)	2 (50)	2 (50)	5 (33)
* Severe*	0	2 (50)	0	2 (13)
Collagen alterations, n (%)				
* Mild*	4 (100)	2 (50)	2 (50)	4 (29)
* Moderate*	0	2 (50)	2 (50)	9 (64)
* Severe*	0	0	0	2 (14)

*Abbreviations*: *AAOCA* anomalous aortic origin of the coronary artery, *MEMA* mucoid extracellular matrix accumulation, *SMC* smooth muscle cells

*In 1 control the presence of nuclei was inconclusive and in all controls assessment of MEMA was inconclusive

**Fig. 7:**
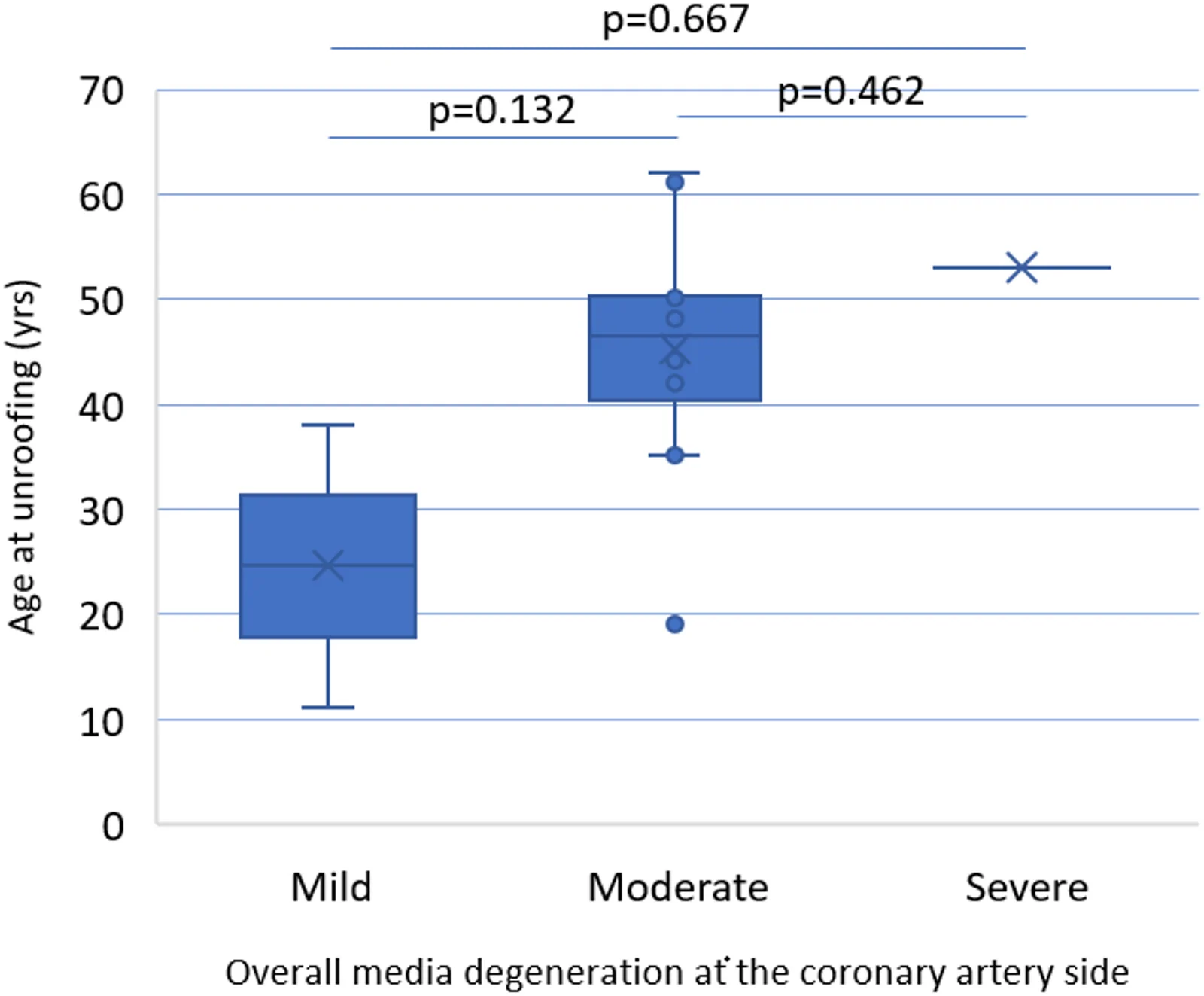
Bar graph illustrating the median age per category of media degeneration of the tunica media of the intramural segment at the coronary arterial side

As media degeneration is known to be progressive with age, results were stratified by patient age. Although not statistically significant, a trend was observed indicating an association between the patients age and the severity of medial degeneration, Fig. 7.

## Discussion

In this multicenter prospective cohort study, using unique intramural segment specimens from pediatric and adult patients with AAOCA, we analyzed the histological characteristics of the intramural segment in relation to vascular wall compliance and their correlation with the aortic commissure/tubular aorta, thereby providing new insights into its potential contribution to the functional consequences of AAOCA.

### A shared tunica media between aorta and AAOCA

This study confirms the definition of an intramural AAOCA as having a shared tunica media of the anomalous coronary artery and aorta, without interposing tunica adventitia. Furthermore, these findings expand this definition by demonstrating that the shared vascular wall may occur between the anomalous coronary artery and either the tubular aorta or the aortic sinus, depending on the AAOCA origin. In addition, we did observe 2 components of this shared medial layer: one on the aortic side and one on the coronary arterial side, which, as such, has not been reported in previous studies. In addition, we found the aortic side and coronary arterial side of the intramural segment to each possess distinguishing characteristics.

### Aortic valve commissure on the aortic side of the intramural segment

The tunica media on the aortic sinus/proximal tubular aortic side revealed predominantly a band-like layer without smooth muscle cells and more pronounced fibrous tissue, histologically resembling aortic valve commissure. This was supported by the narrow spatial relation of the AAOCA ostium to the aortic valve commissure on pre-operative CTAs, and by the inverse relation between the distance of the AAOCA ostium to the aortic valve commissural tissue and the amount of fibrous tissue (*R*=-0.55, *p* = 0.04). Therefore, we consider the observed vascular characteristics on the aortic side of the intramural segment to be aortic valve commissural tissue.

### Vascular wall pathology at the coronary arterial side of the intramural segment

In contrast, the vascular wall characteristics in the intramural segment on the coronary arterial artery side, were characterized by increased glycoprotein accumulation, elastic fiber fragmentation and/or loss, elastic fiber thinning, smooth muscle cell nuclei loss, and collagen alterations. Some of these features are also present in normal aortic sinus tissue [20]; however, as the characteristics were shown particularly on the coronary arterial side of the intramural segment, these findings more likely reflect vascular wall pathology and/or remodeling processes [20].

### Findings in relation to literature

Data on the histological features of the intramural segment in adult patients with AAOCA are scarce and, if present, very limited. To evaluate our findings in light of current knowledge a literature search was performed (Figure S3). Ten studies were found that included some form of histological assessment of the intramural segment of AAOCA, Table 6 [4, 7–10, 21–25]. In line with findings in the current study, each article stated that the intramural segment courses through the tunica media without interference from a tunica adventitia. In two articles by Hata et al. and Carreon et al. [24, 25] further specification of the histological features of the intramural segment was provided. Hata et al. histologically assessed 5 post-mortem AAORCA specimens and described that the elastic fibers and a few smooth muscle cells are the main components of the intramural segment, suggesting a difference from an epicardial coronary artery, in which smooth muscle cells are more prevalent [24]. Carreon et al. investigated the intramural segments of 20 AAOCA (4 AAOLCA and 16 AAORCA, age range 7–18 years) specimens post-unroofing procedure [25]. Like in the current study, various vascular wall alterations, e.g. vascular wall fibrosis, smooth muscle disarray and hyperplasia, elastic fiber disorganization and fragmentation, and extracellular mucoid matrix accumulation, were observed. This study found no relation between these vascular characteristics and CTA parameters, such as distance to the aortic valve commissure, however, similar characteristics in the aortic valve commissures of normal heart specimens were observed. The study did observe a trend toward more severe mural fibrosis and elastic fiber alterations in patients > 10 years of age at the time of surgery, although this was not statistically significant, and no patients > 18 years of age were included.

**Table 6:** Results of literature search on histological characteristics of the tunica media in anomalous coronary arteries

Author and year	(*n*) AAOCA	Age	Gender	Examination	Histological statements and/or conclusions concerning the intramural course
Gittenberger-de Groot et al. 1986 [7]	2 AAOLCA and 1 AAORCA in TGA patients	Range 1.5–8.5 yrs	2 males, 1 female	Post-mortem microscopy	“The media of the aorta and coronary artery are continuous. There is no intervening loose fibrous tissue of the adventitia (AD) separating the elastic lamellae. (van Gieson elastic tissue stain.)”
Corrado et al. 1990 [21]	2 AAORCA	22 and 29 yrs	2 males	Post-mortem microscopy	“The proximal tract of the right coronary artery had a slit-like lumen, and was sandwiched between the aorta and the pulmonary infundibulum within the aortic tunica media.”
Frescura et al. 1998 [10]	4 AAOLCA and 7 AAORCA	22 yrs, 10 unknown	1 male, 10 unknown	Post-mortem microscopy	“In all cases of RCA origin from left aortic sinus and LCA from right aortic sinus, the proximal tract of the abnormal coronary artery ran between the aorta and the pulmonary infundibulum with an initial intramural course within the aortic tunica media and a slit-like lumen.”
Basso et al. 2000 [4]	2 AAOLCA and 1 AAORCA	15 yrs, 2 unknown	1 male, 2 unknown	Post-mortem microscopy	“In three of these hearts, based on histologic examination, the course of the proximal portion of the anomalous coronary artery (LMCA in two, RCA in one) was documented to be intramural, i.e., contained completely within the aortic wall, so that the coronary artery and the aorta shared the same media without an interpositioned adventitia.”
Angelini et al. 2018 [9]	1 AAOLCA	Unknown	Unknown	Post-mortem microscopy	“The segment travelling to the left side has an intramural course inside the aortic tunica media”
Tomanek et al. 2019 [22]	1 AAOLCA	Unknown	Unknown	Post-mortem microscopy	“IM proximal course runs consistently inside the aortic tunica media”
Koppel et al. 2022 [8]	1 AAORCA	Unknown	Unknown	Post-mortem microscopy	“An intramural course is histologically defined by a shared tunica media (without interposing adventitia) of the aorta and the coronary artery”
Angelini et al. 2023 [23]	1 AAOLCA and 1 AAORCA	14 and 17 yrs	1 male, 1 female	Post-mortem microscopy	“The IM segment is made of aortic wall covered by coronary endothelium” “The IM variety features intramural proximal segment of the initial section (inside the aortic media, at the level of and parallel to the sinutubular junction, adjacent to the aortic root) with dynamic, variable stenosis.”
Hata et al. 2015 [24]	5 AAORCA	Range 22–75 yrs	5 males	Post-mortem microscopy	“In all of the cases, parts of the wall of the intramural RCA and aorta shared a common media, as shown histologically. There was no interpositioned adventitia.” “Immunohistochemistry using α-smooth muscle actin also showed a small amount of smooth muscle cells in the media of the intramural segment of the RCA, and CD 34 showed continuous lining of endothelial cells of the luminal surface.” “Anatomically, the medial layer of the intramural segment of RCA is mainly composed of elastic fibers, as well as a few smooth muscle cells. This feature of the intramural region of RCA is different to that of the epicardial coronary artery, which has smooth muscle as the major component of the media.”
Carreon et al. 2023 [25]	4 AAOLCA and 16 AAORCA	Range 7–18 yrs	14 males, 6 females	Post-unroofing microscopy	“A common medial layer shared by the aorta and the intramural segment of the anomalous coronary artery without any intervening adventitial layer.” “Varying degrees of wall fibrosis, smooth muscle disarray and hyperplasia, elastic fiber disorganization and fragmentation, and extracellular mucoid matrix accumulation in the resected common wall, with the elastic fiber alterations being the most frequent finding.” “These findings could be secondary to the close proximity of the excised segment to a commissure, given the histologic pattern observed in pericommissural areas in the vessel walls of the normal heart.” “The shared wall of the intramural segment is more akin to an altered elastic aortic wall than to a muscular coronary artery wall.”

*Abbreviations*: *AAOCA* anomalous aortic origin of a coronary artery, *AAOLCA* anomalous aortic origin of a left coronary artery, *AAORCA* anomalous aortic origin of a right coronary artery, *IVUS* intravascular ultrasound, *LCA* left coronary artery, *LMCA* left main coronary artery, *RCA* right coronary artery, *TGA* transposition of the great arteries, *yrs* years old

### Findings in relation to normal aortic sinus, tubular aorta and coronary arterial vascular walls

In the normal aortic wall, an elastic artery, the tunica media comprises elastic and collagen fibers, mainly types II and III, and smooth muscle cells, while the adventitia contains fibroblasts, collagen-rich connective tissue, mainly type I collagen, elastin, vasa vasorum, and nerve fibers [26–28]. Notably, the histological organization differs between the tubular aorta (i.e. intrapericardial ascending) and the aortic sinus. The tubular aorta exhibits greater medial thickness, increased lamellar thickness, and a higher density of elastic fibers compared with the aortic sinus [20]. The sinutubular junction represents a transitional anatomical and functional zone between the aortic sinuses and the tubular aorta [29]. In contrast, the coronary artery, a muscular artery, is characterized by more smooth muscle cells, compared to the aortic sinus or tubular aorta, and an external elastic lamina demarcating the transition to the adventitia [30]. In our control specimens, the histological architecture of the aortic sinus and coronary arterial walls—including intima, media, and adventitia—was comparable to that described in normal aortic sinus and coronary arterial vascular anatomy, respectively. At the aorta–coronary artery junction, tissue characteristics resembled both vessel types; however, the elastic lamellae showed multidirectional directions, likely reflecting differences in wall orientations between the aortic sinus and coronary artery. The thickness of the elastic lamellae also gradually decreased from the aortic sinus toward the coronary artery.

### Findings as related to origin of the anomalous coronary artery with respect to the aortic valve commissure

All patients in our cohort exhibited juxtacommissural coronary arterial origins. The aortic valve commissure is a distinct structure of the aortic sinus vascular wall, composed predominantly of fibrous tissue that provides structural support to the aortic valve leaflets. In the post-mortem AAOCA specimen, this commissural tissue was characterized, and comparable features were identified in particular on the aortic side of the intramural segment of the prospectively collected AAOCA specimens. Based on these observations, the tissue in question most likely represents aortic valve commissure rather than aortic vascular wall tissue. This pattern was consistently observed across all included AAOCA specimens. Notably, these characteristics of aortic valve commissural tissue were also observed in AAOCA specimens in which the AAOCA originated from above the STJ: 12 patients (80%) had an coronary arterial ostium located at or below the STJ (7 at the level of the STJ and 5 below), whereas in 3 patients (20%) the ostium was located above the STJ (i.e. AAOCA ostium from the tubular aorta), as summarized in Table 3. The maximal observed distances ranged from 2 mm below to 3–4 mm above the STJ, as estimated by computed tomography angiography (CTA) and illustrated in Fig. 6B. One possible explanation, given that this finding was also observed in AAOCA origins located just above or below the STJ, is that the aortic valve commissure, which includes the upward extensions of the valvular “crown”, may histologically extend further into the aortic wall at the level of the STJ, or even slightly above it, where it gradually merges with the surrounding tubular aorta. This interpretation is consistent with the observation that the coronary arteries originated in close proximity to the STJ. This is also in line with the inverse correlation of the amount of fibrous tissue in the intramural segment of coronary arteries originating more distal from the STJ (i.e. gradual decrease in fibrous tissue amount more distal from the STJ). Alternatively, the apparent discrepancy may reflect the limited spatial accuracy of CTA-based measurements of the distance between the AAOCA origin and the STJ, as compared with direct histological assessment.

### Relevance of vascular abnormalities on coronary arterial side of the intramural segment

Vascular wall abnormalities were consistently observed on the coronary arterial side of the intramural segment, distinct from the aortic valve commissural tissue predominantly located on the aortic side. These abnormalities included increased glycoprotein accumulation, elastic fiber fragmentation and/or loss, elastic fiber thinning, loss of smooth muscle cell nuclei, and alterations in collagen architecture. In contrast to a normal tubular aorta, normal aortic sinus tissue exhibits reduced medial and lamellar thickness, as well as a more non-parallel organization of elastic fibers [20]. Histological variation is also present within the aortic sinus itself. Notably, several features of the normal aortic sinus tissue overlap with those commonly described as medial degeneration [20]. Given that certain features were predominantly observed on the coronary arterial side of the intramural septum, the possibility that they indicate coronary arterial vascular wall remodeling is more plausible. Such remodeling could result from altered hemodynamic stress within the intramural segment due to the anomalous coronary anatomy. Cardiac MRI, especially 4D flow imaging, might offer a future path to clarify how intramural hemodynamic stress drives vascular wall remodeling [31]. However, vascular remodeling is typically characterized by smooth muscle cell hyperplasia, adventitial remodeling and/or intimal thickening, none of which were observed to a significant extent in the present data. In contrast, the observed mucoid extracellular matrix accumulation, elastic fiber fragmentation and loss, and loss of smooth muscle cell nuclei are more consistent with medial degeneration [18]. This is also supported by the fact that certain features tend to increase with age, which could be more reflective of medial degeneration. Additionally, the absence of vasa vasorum and nervorum in the intramural segment may limit an adequate autonomous self-healing response of the cells, thereby further promoting medial degeneration rather than structural remodeling.

###  Relevance of findings on sudden cardiac risk?

Current clinical guidelines do not consider the distance between the AAOCA ostium and the aortic valve commissure as a factor in SCD risk stratification. In our cohort, the AAOCA ostium was consistently located in close proximity to the aortic valve commissure. Although none of these patients experienced SCD, this spatial relationship might be relevant. Hata et al. [24] reported a 32-year-old patient with AAORCA who suffered SCD and showed a greater ostium–commissure distance, raising the hypothesis that aortic valve commissural proximity might influence risk. Mechanistically, luminal collapse of the AAOCA requires lower intraluminal pressure relative to surrounding tissue. A proximal obstruction—such as a valve-like ridge—could promote this pressure drop and predispose to compression. Aortic valve commissural tissue, being more rigid and fibrous than the regular vascular wall, is unlikely to form such a ridge and may therefore protect against obstruction. Furthermore, since most coronary arterial flow occurs during diastole when the aortic valve is closed, a valve acommissure in close proximity could stretch the intramural segment toward the aortic lumen, potentially enhancing coronary arterial filling. This effect may be attenuated when the ostium lies farther from the valve commissure. As all patients in the present study survived, further investigation, including post-SCD cases, is warranted to clarify this potential relationship.

### Limitations

This study assessed the histological features of the intramural segments of a small group of patients who underwent the unroofing procedure. Due to the differing origins of the tissue, the duration of fixation and preservation was not entirely uniform across the prospectively included specimens, control specimens, and post-mortem specimen. Although it is unlikely that this variability substantially influenced the histological analyses, it may theoretically have introduced minor differences in the results. Furthermore, the study population reflects the low prevalence of AAOCA, the small number of patients included should be taken into account when interpreting the outcomes. None of the patients presented with acute myocardial ischemia or (non-fatal) cardiac arrest, therefore, survivorship bias cannot be excluded.

### Conclusion and clinical implications

This histological study of patients with AAOCA demonstrated that the anomalous coronary artery courses through the tunica media of the aortic sinus or tubular aorta without an interposing adventitia, forming two distinct medial layers. The layer on the aortic side demonstrates features consistent with aortic valve commissural tissue, supported by its histological composition and close spatial relationship to the aortic valve commissure. In contrast, the pronounced abnormalities observed on the coronary arterial side of the segment likely reflect intrinsic vascular wall pathology—such as medial degeneration or remodeling. These insights expand current understanding of AAOCA morphology and open avenues for further exploration to improve risk stratification. Future histological studies comparing benign AAOCA cases with specimens from patients who experienced acute myocardial infarction or SCD are warranted to clarify the full pathophysiological spectrum of AAOCA.

## Supplementary Information


Additional file 1: Supplemental Figure S1. Unroofing procedure. Supplemental Figure S2. Graph illustrating the distribution of age at initial diagnosis of AAOCA of the included specimen. Supplemental Figure S3. Prisma Flow diagram showing the search, screening and inclusion of literature on histological characteristics of the tunica media in anomalous coronary arteries. Supplemental Table S1. Outcomes of quantitative grading of the tunica media of the prospectively included specimens. Supplemental Text S1. Score list for quantitative grading of the tunica media of the AAOCA specimens. Supplemental Text S2. Search strategy for histological characteristics of the tunica media in anomalous coronary arteries.



Additional file 2: Supplemental Video S1. A 3D reconstruction of computed tomography imaging from pre-unroofing to post-unroofing.


## Data Availability

The data underlying this article will be shared upon reasonable request to the corresponding author.

## Abbreviations

α-SMA: α-Smooth Muscle Actin, clone 1A4
AAOCA: Anomalous aortic origin of a coronary artery
AAOLCA: Anomalous aortic origin of a left coronary artery
AAORCA: Anomalous aortic origin of a right coronary artery
ACHD: Adults with Congenital Heart Disease
CTA: Computed tomography angiography
H&E: Hematoxylin & Eosin
ILS: Intraluminal space
MEMA: Mucoid extracellular matrix accumulation
Movat: Movats pentachrome
MRI: Magnetic resonance imaging
PECAM-1: Platelet endothelial cell adhesion molecule-1
PFA: Paraformaldehyde
RF: Resorcin fuchsin
SCD: Sudden cardiac death
SR: Sirius Red
STJ: Sinutubular junction
Tub3: βIII-tubulin
