# Supplementary material for: Histological assessment of the intramural segment in adults with an anomalous aortic origin of a coronary artery with an interarterial course
Source: BMC Cardiovasc Disord. 2026 Jun 19;26:760. doi: 10.1186/s12872-026-06000-7 (PMC13531909; doi:10.1186/s12872-026-06000-7)
Supplement: Supplementary file 1 [file 12872_2026_6000_MOESM1_ESM.docx]

**Supplemental Figure 1.** Unroofing procedure.

Abbreviations: AAORCA, anomalous aortic origin of a right coronary artery; AoAsc, ascending aorta

**Supplemental Figure 2.** Graph illustrating the distribution of age at initial diagnosis of AAOCA of the included specimen.

Age at initial diagnosis of AAOCA (years)

**Supplemental Figure 3.** Prisma Flow diagram showing the search, screening and inclusion of literature on histological characteristics of the shared vascular wall in anomalous coronary arteries.

Abbreviations: AAOCA, anomalous aortic origin of a coronary artery; ALCAPA, anomalous left coronary artery from the pulmonary artery.

**Supplemental Table 1.** Outcomes of quantitative grading of the tunica media of the prospectively included specimens.

|  | **Coronary artery side n=15** |
| --- | --- |
| **Overall medial degeneration, n (%)** | |
| Mild | 2 (13)  12 (80)  1 (7) |
| Moderate |  |
| Severe |  |
| **Mucoid ECM accumulation, n (%)** | |
| Intralamellar | |
| Grade | |
| Mild | 0  2 (13)  0 |
| Moderate |  |
| Severe |  |
| Distribution | |
| Focal | 1 (7)  1 (7)  0 |
| Multifocal |  |
| Extensive |  |
| Translamellar | |
| Grade | |
| Mild | 2 (13)  8  3 (20) |
| Moderate |  |
| Severe |  |
| Distribution | |
| Focal | 1 (7)  9 (60)  3 (20) |
| Multifocal |  |
| Extensive |  |
| **Elastic fiber fragmentation and/or loss, n (%)** | |
| Grade | |
| Absent | 1 (7)  2 (13)  8 (53)  4 (27) |
| Mild |  |
| Moderate |  |
| Severe |  |
| Distribution | |
| Absent | 1 (7)  1 (7)  8 (53)  5 (33) |
| Focal |  |
| Multifocal |  |
| Extensive |  |
| **Elastic fiber thinning, n (%)** | |
| Grade | |
| Mild | 4 (27)  6 (40)  5 (33) |
| Moderate |  |
| Severe |  |
| Distribution | |
| Focal | 1 (7)  7 (47)  7 (47) |
| Multifocal |  |
| Extensive |  |
| **Elastic fiber disorganization, n (%)** | |
| Distribution | |
| Focal | 1 (7)  7 (47)  7 (47) |
| Multifocal |  |
| Extensive |  |
| **SMC nuclei loss, n (%)** | |
| Type | |
| Patchy | 14 (93)  1 (7) |
| Band-like |  |
| Distribution | |
| Rare | 10 (67)  3 (20)  2 (13) |
| Frequent |  |
| Extensive |  |
| **Laminar medial collapse, n (%)** | |
| Appearance | |
| Thin | 13 (87)  0  2 (13) |
| Dense |  |
| Broken |  |
| Distribution | |
| Focal | 8 (53)  5 (33)  2 (13) |
| Multifocal |  |
| Extensive |  |
| **SMC disorganization, n (%)** | |
| Distribution | |
| Absent | 0  10 (67)  5 (33) |
| Focal |  |
| Multifocal |  |
| **Medial** **fibrosis, n (%)** | |
| Intralamellar | |
| Grade | |
| Mild | 3 (20)  3 (20)  0 |
| Moderate |  |
| Severe |  |
| Distribution | |
| Focal | 3 (20)  3 (20)  0 |
| Multifocal |  |
| Extensive |  |
| Translamellar | |
| Grade | |
| Mild | 2 (13)  6 (40)  1 (7) |
| Moderate |  |
| Severe |  |
| Distribution | |
| Focal | 1 (7)  7 (47)  1 (7) |
| Multifocal |  |
| Extensive |  |

**Supplemental Text 1.** Score list for quantitative grading of the tunica media of the AAOCA specimens.

MID SEGMENT 1:

1. MEMA
   a. Intralamellar MEMA-I
      i. Grade	Mild/moderate/severe
      ii. Distribution	Absent/focal/multifocal/extensive
   b. Translamellar MEMA-T
      i. Grade	Mild/moderate/severe
      ii. Distribution	Absent/focal/multifocal/extensive
2. Elastic fiber alterations
   a. Elastic fiber fragmentation and/or loss
      i. Grade — mild/moderate/severe.
      ii. Distribution — absent/focal/multifocal/extensive.
   b. Elastic fiber thinning
      i. Grade — mild/moderate/severe.
      ii. Distribution — absent/focal/multifocal/extensive
   c. Elastic fiber disorganization
      i. Distribution — absent/focal/multifocal/extensive
3. Smooth muscle cell alterations
   a. Smooth muscle cell nuclei loss
      i. Distribution — absent/rare/frequent/extensive.
      ii. Type — patchy/band-like
   b. Laminar medial collapse
      i. Appearance — thin/dense/broken
      ii. Distribution — absent/focal/multifocal/extensive
   c. Smooth muscle cell disorganization
      i. Distribution — focal/multifocal/extensive
4. Collagen alterations
   a. Medial fibrosis
      i. Intralamellar (I): the increase in collagen does not significantly alter the arrangement of the lamellar units
         1. Grade — mild/moderate/severe.
         2. Distribution — absent/focal/multifocal/extensive
      ii. Translamellar (T): the increase in collagen is more scar-like altering the arrangement of the lamellar units
          1. Grade — mild/moderate/severe.
          2. Distribution — absent/focal/multifocal/extensive
5. Overall medial degeneration
   a. The degree of severity is rated as follows:
      i. Mild: lesion involves up to 3 lamellar units.
      ii. Moderate: lesion involves 4–10 lamellar units.
      iii. Severe: lesion involves N10 lamellar units.
   b. The extent of individual lesions is graded as follows:
      i. Absent: the lesion type is not present.
      ii. Focal: involvement of the media area up to 10%.
      iii. Multifocal: multiple areas of involvement of N10–30% of the media area.
      iv. Extensive: involvement of the media area N30%.

MID SEGMENT 2:

1. MEMA
   a. Intralamellar MEMA-I
      i. Grade	Mild/moderate/severe
      ii. Distribution	Absent/focal/multifocal/extensive
   b. Translamellar MEMA-T
      i. Grade	Mild/moderate/severe
      ii. Distribution	Absent/focal/multifocal/extensive
2. Elastic fiber alterations
   a. Elastic fiber fragmentation and/or loss
      i. Grade — mild/moderate/severe.
      ii. Distribution — absent/focal/multifocal/extensive.
   b. Elastic fiber thinning
      i. Grade — mild/moderate/severe.
      ii. Distribution — absent/focal/multifocal/extensive
   c. Elastic fiber disorganization
      i. Distribution — absent/focal/multifocal/extensive
3. Smooth muscle cell alterations
   a. Smooth muscle cell nuclei loss
      i. Distribution — absent/rare/frequent/extensive.
      ii. Type — patchy/band-like
   b. Laminar medial collapse
      i. Appearance — thin/dense/broken
      ii. Distribution — absent/focal/multifocal/extensive
   c. Smooth muscle cell disorganization
      i. Distribution — focal/multifocal/extensive
4. Collagen alterations
   a. Medial fibrosis
      i. Intralamellar (I): the increase in collagen does not significantly alter the arrangement of the lamellar units
         1. Grade — mild/moderate/severe.
         2. Distribution — absent/focal/multifocal/extensive
      ii. Translamellar (T): the increase in collagen is more scar-like altering the arrangement of the lamellar units
          1. Grade — mild/moderate/severe.
          2. Distribution — absent/focal/multifocal/extensive
5. Overall medial degeneration
   a. The degree of severity is rated as follows:
      i. Mild: lesion involves up to 3 lamellar units.
      ii. Moderate: lesion involves 4–10 lamellar units.
      iii. Severe: lesion involves N10 lamellar units.
   b. The extent of individual lesions is graded as follows:
      i. Absent: the lesion type is not present.
      ii. Focal: involvement of the media area up to 10%.
      iii. Multifocal: multiple areas of involvement of N10–30% of the media area.
      iv. Extensive: involvement of the media area N30%.

**Supplementary Text S2.** Literature search strategy on histology of the tunica media in anomalous coronary arteries

Databases

**PubMed**

<http://www.ncbi.nlm.nih.gov/pubmed?otool=leiden>

Anomalous coronary arteries & intramural course

(("Anomalous aortic origin of a coronary artery"[tw] OR "Anomalous aortic origin of coronary artery"[tw] OR "Anomalous aortic origin of coronary arteries"[tw] OR "Anomalous aortic origin of the coronary artery" [tw] OR "Anomalous aortic origin of the coronary arteries"[tw] OR "Anomalous aortic origin of the left coronary artery" [tw] OR "Anomalous aortic origin of the right coronary artery"[tw] OR "AAOCA"[tw] OR "Anomalous coronary artery from the opposite sinus of Valsalva"[tw] OR "anomalous coronary arteries originating from the opposite sinus of valsalva"[tw] OR "anomalous coronary artery originating from the opposite sinus of valsalva"[tw] OR "ACAOS"[tw] OR "Anomalous coronary artery"[tw] OR "Anomalous coronary arteries"[tw] OR "Anomalous coronary arterial"[tw] OR "Coronary anomaly"[tw] OR "Coronary anomalies"[tw] OR "Coronary anomalous"[tw] OR "Coronary artery anomaly"[tw] OR "Coronary artery anomalies"[tw] OR "Coronary artery anomalous"[tw] OR "aberrant coronary arteries"[tw] OR "aberrant coronary artery"[tw] OR "aberrant coronary"[tw] OR "Coronary Vessel Anomalies"[Mesh:NoExp] OR "aberrant coronary arteries"[title/abstract:~6] OR "aberrant coronary artery"[title/abstract:~6] OR "aberrant coronary"[title/abstract:~6] OR "Anomalies aorta origin coronary arteries"[title/abstract:~6] OR "Anomalies aorta origin coronary artery"[title/abstract:~6] OR "Anomalies aortic origin coronary arteries" [title/abstract:~6] OR "Anomalies aortic origin coronary artery"[title/abstract:~6] OR "Anomalies aortic origin left coronary artery" [title/abstract:~6] OR "Anomalies aortic origin right coronary artery"[title/abstract:~6] OR "Anomalies coronary arterial"[title/abstract:~6] OR "anomalies coronary arteries originating opposite sinus valsalva"[title/abstract:~6] OR "Anomalies coronary arteries"[title/abstract:~6] OR "Anomalies coronary artery opposite sinus Valsalva"[title/abstract:~6] OR "anomalies coronary artery originating opposite sinus valsalva"[title/abstract:~6] OR "Anomalies coronary artery"[title/abstract:~6] OR "Anomalous aorta origin coronary arteries"[title/abstract:~6] OR "Anomalous aorta origin coronary artery"[title/abstract:~6] OR "Anomalous aortic origin coronary arteries" [title/abstract:~6] OR "Anomalous aortic origin coronary artery"[title/abstract:~6] OR "Anomalous aortic origin left coronary artery" [title/abstract:~6] OR "Anomalous aortic origin right coronary artery"[title/abstract:~6] OR "Anomalous coronary arterial"[title/abstract:~6] OR "anomalous coronary arteries originating opposite sinus valsalva"[title/abstract:~6] OR "Anomalous coronary arteries"[title/abstract:~6] OR "Anomalous coronary artery opposite sinus Valsalva"[title/abstract:~6] OR "anomalous coronary artery originating opposite sinus valsalva"[title/abstract:~6] OR "Anomalous coronary artery"[title/abstract:~6] OR "Anomaly aorta origin coronary arteries"[title/abstract:~6] OR "Anomaly aorta origin coronary artery"[title/abstract:~6] OR "Anomaly aortic origin coronary arteries" [title/abstract:~6] OR "Anomaly aortic origin coronary artery"[title/abstract:~6] OR "Anomaly aortic origin left coronary artery" [title/abstract:~6] OR "Anomaly aortic origin right coronary artery"[title/abstract:~6] OR "Anomaly coronary arterial"[title/abstract:~6] OR "anomaly coronary arteries originating opposite sinus valsalva"[title/abstract:~6] OR "Anomaly coronary arteries"[title/abstract:~6] OR "Anomaly coronary artery opposite sinus Valsalva"[title/abstract:~6] OR "anomaly coronary artery originating opposite sinus valsalva"[title/abstract:~6] OR "Anomaly coronary artery"[title/abstract:~6] OR "Coronary anomalies"[title/abstract:~6] OR "Coronary anomalous"[title/abstract:~6] OR "Coronary anomaly"[title/abstract:~6] OR "Coronary artery anomalies"[title/abstract:~6] OR "Coronary artery anomalous"[title/abstract:~6] OR "Coronary artery anomaly"[title/abstract:~6]) AND ("Intramural"[tw] OR "Intra mural"[tw] OR "Intramural*"[tw] OR "Intra mural*"[tw]))

AND ("Tunica Media"[Mesh] OR "Tunica media"[tw] OR "Tunica media*"[tw] OR "Arterial Media"[tw] OR "Vascular Media"[tw] OR "Venous Media"[tw] OR "Tunica"[tw] OR "Tunica*"[tw] **OR "Vascular Smooth Muscle"[tw] OR "Vascular Smooth Muscles"[tw]** OR "Adventitia"[Mesh] OR "Tunica adventitia"[tw] OR "Adventitial Tissue"[tw] OR "Adventitial Tissues"[tw] OR "Adventitious Tissue"[tw] OR "Adventitious Tissues"[tw] OR "Tunica Externa"[tw] OR "Adventitia"[tw] OR "Adventiti*"[tw] OR "Periadventitia"[tw] OR "Periadventiti*"[tw] **OR "Media"[tiab]**))

**PubMed Central**

<https://www.ncbi.nlm.nih.gov/pmc/>

Anomalous coronary arteries & intramural course & Tunica Media

(coronary[title] AND ("Anomalous aortic origin of a coronary artery"[tw] OR "Anomalous aortic origin of coronary artery"[tw] OR "Anomalous aortic origin of coronary arteries"[tw] OR "Anomalous aortic origin of the coronary artery" [tw] OR "Anomalous aortic origin of the coronary arteries"[tw] OR "Anomalous aortic origin of the left coronary artery" [tw] OR "Anomalous aortic origin of the right coronary artery"[tw] OR "AAOCA"[tw] OR "Anomalous coronary artery from the opposite sinus of Valsalva"[tw] OR "anomalous coronary arteries originating from the opposite sinus of valsalva"[tw] OR "anomalous coronary artery originating from the opposite sinus of valsalva"[tw] OR "ACAOS"[tw] OR "Anomalous coronary artery"[tw] OR "Anomalous coronary arteries"[tw] OR "Anomalous coronary arterial"[tw] OR "Coronary anomaly"[tw] OR "Coronary anomalies"[tw] OR "Coronary anomalous"[tw] OR "Coronary artery anomaly"[tw] OR "Coronary artery anomalies"[tw] OR "Coronary artery anomalous"[tw] OR "aberrant coronary arteries"[tw] OR "aberrant coronary artery"[tw] OR "aberrant coronary"[tw] OR "Coronary Vessel Anomalies"[Mesh:NoExp] OR "aberrant coronary arteries"[title/abstract:~6] OR "aberrant coronary artery"[title/abstract:~6] OR "aberrant coronary"[title/abstract:~6] OR "Anomalies aorta origin coronary arteries"[title/abstract:~6] OR "Anomalies aorta origin coronary artery"[title/abstract:~6] OR "Anomalies aortic origin coronary arteries" [title/abstract:~6] OR "Anomalies aortic origin coronary artery"[title/abstract:~6] OR "Anomalies aortic origin left coronary artery" [title/abstract:~6] OR "Anomalies aortic origin right coronary artery"[title/abstract:~6] OR "Anomalies coronary arterial"[title/abstract:~6] OR "anomalies coronary arteries originating opposite sinus valsalva"[title/abstract:~6] OR "Anomalies coronary arteries"[title/abstract:~6] OR "Anomalies coronary artery opposite sinus Valsalva"[title/abstract:~6] OR "anomalies coronary artery originating opposite sinus valsalva"[title/abstract:~6] OR "Anomalies coronary artery"[title/abstract:~6] OR "Anomalous aorta origin coronary arteries"[title/abstract:~6] OR "Anomalous aorta origin coronary artery"[title/abstract:~6] OR "Anomalous aortic origin coronary arteries" [title/abstract:~6] OR "Anomalous aortic origin coronary artery"[title/abstract:~6] OR "Anomalous aortic origin left coronary artery" [title/abstract:~6] OR "Anomalous aortic origin right coronary artery"[title/abstract:~6] OR "Anomalous coronary arterial"[title/abstract:~6] OR "anomalous coronary arteries originating opposite sinus valsalva"[title/abstract:~6] OR "Anomalous coronary arteries"[title/abstract:~6] OR "Anomalous coronary artery opposite sinus Valsalva"[title/abstract:~6] OR "anomalous coronary artery originating opposite sinus valsalva"[title/abstract:~6] OR "Anomalous coronary artery"[title/abstract:~6] OR "Anomaly aorta origin coronary arteries"[title/abstract:~6] OR "Anomaly aorta origin coronary artery"[title/abstract:~6] OR "Anomaly aortic origin coronary arteries" [title/abstract:~6] OR "Anomaly aortic origin coronary artery"[title/abstract:~6] OR "Anomaly aortic origin left coronary artery" [title/abstract:~6] OR "Anomaly aortic origin right coronary artery"[title/abstract:~6] OR "Anomaly coronary arterial"[title/abstract:~6] OR "anomaly coronary arteries originating opposite sinus valsalva"[title/abstract:~6] OR "Anomaly coronary arteries"[title/abstract:~6] OR "Anomaly coronary artery opposite sinus Valsalva"[title/abstract:~6] OR "anomaly coronary artery originating opposite sinus valsalva"[title/abstract:~6] OR "Anomaly coronary artery"[title/abstract:~6] OR "Coronary anomalies"[title/abstract:~6] OR "Coronary anomalous"[title/abstract:~6] OR "Coronary anomaly"[title/abstract:~6] OR "Coronary artery anomalies"[title/abstract:~6] OR "Coronary artery anomalous"[title/abstract:~6] OR "Coronary artery anomaly"[title/abstract:~6]) AND ("Intramural"[tw] OR "Intra mural"[tw] OR "Intramural*"[tw] OR "Intra mural*"[tw]) AND ("Tunica Media"[Mesh] OR "Tunica media"[tw] OR "Tunica media*"[tw] OR "Arterial Media"[tw] OR "Vascular Media"[tw] OR "Venous Media"[tw] OR "Tunica"[tw] OR "Tunica*"[tw] **OR "Vascular Smooth Muscle"[tw] OR "Vascular Smooth Muscles"[tw]** OR "Adventitia"[Mesh] OR "Tunica adventitia"[tw] OR "Adventitial Tissue"[tw] OR "Adventitial Tissues"[tw] OR "Adventitious Tissue"[tw] OR "Adventitious Tissues"[tw] OR "Tunica Externa"[tw] OR "Adventitia"[tw] OR "Adventiti*"[tw] OR "Periadventitia"[tw] OR "Periadventiti*"[tw] **OR "Media"[tw]**))

**Embase**

<http://ovidsp.ovid.com/ovidweb.cgi?T=JS&PAGE=main&MODE=ovid&D=oemezd>

Anomalous coronary arteries & intramural course

(("Anomalous aortic origin of a coronary artery".mp OR "Anomalous aortic origin of coronary artery".mp OR "Anomalous aortic origin of coronary arteries".mp OR "Anomalous aortic origin of the coronary artery" .mp OR "Anomalous aortic origin of the coronary arteries".mp OR "Anomalous aortic origin of the left coronary artery" .mp OR "Anomalous aortic origin of the right coronary artery".mp OR "AAOCA".mp OR "Anomalous coronary artery from the opposite sinus of Valsalva".mp OR "anomalous coronary arteries originating from the opposite sinus of valsalva".mp OR "anomalous coronary artery originating from the opposite sinus of valsalva".mp OR "ACAOS".mp OR "Anomalous coronary artery".mp OR "Anomalous coronary arteries".mp OR "Anomalous coronary arterial".mp OR "Coronary anomaly".mp OR "Coronary anomalies".mp OR "Coronary anomalous".mp OR "Coronary artery anomaly".mp OR "Coronary artery anomalies".mp OR "Coronary artery anomalous".mp OR "aberrant coronary arteries".mp OR "aberrant coronary artery".mp OR "aberrant coronary".mp OR "Coronary Artery Anomaly"/ OR (("aberrant" ADJ6 "coronary" ADJ6 "arteries") OR ("aberrant" ADJ6 "coronary" ADJ6 "artery") OR ("aberrant" ADJ6 "coronary") OR ("Anomalies" ADJ6 "aorta" ADJ6 "origin" ADJ6 "coronary" ADJ6 "arteries") OR ("Anomalies" ADJ6 "aorta" ADJ6 "origin" ADJ6 "coronary" ADJ6 "artery") OR ("Anomalies" ADJ6 "aortic" ADJ6 "origin" ADJ6 "coronary" ADJ6 "arteries"" ADJ6 ") OR ("Anomalies" ADJ6 "aortic" ADJ6 "origin" ADJ6 "coronary" ADJ6 "artery") OR ("Anomalies" ADJ6 "aortic" ADJ6 "origin" ADJ6 "left" ADJ6 "coronary" ADJ6 "artery"" ADJ6 ") OR ("Anomalies" ADJ6 "aortic" ADJ6 "origin" ADJ6 "right" ADJ6 "coronary" ADJ6 "artery") OR ("Anomalies" ADJ6 "coronary" ADJ6 "arterial") OR ("anomalies" ADJ6 "coronary" ADJ6 "arteries" ADJ6 "originating" ADJ6 "opposite" ADJ6 "sinus" ADJ6 "valsalva") OR ("Anomalies" ADJ6 "coronary" ADJ6 "arteries") OR ("Anomalies" ADJ6 "coronary" ADJ6 "artery" ADJ6 "opposite" ADJ6 "sinus" ADJ6 "Valsalva") OR ("anomalies" ADJ6 "coronary" ADJ6 "artery" ADJ6 "originating" ADJ6 "opposite" ADJ6 "sinus" ADJ6 "valsalva") OR ("Anomalies" ADJ6 "coronary" ADJ6 "artery") OR ("Anomalous" ADJ6 "aorta" ADJ6 "origin" ADJ6 "coronary" ADJ6 "arteries") OR ("Anomalous" ADJ6 "aorta" ADJ6 "origin" ADJ6 "coronary" ADJ6 "artery") OR ("Anomalous" ADJ6 "aortic" ADJ6 "origin" ADJ6 "coronary" ADJ6 "arteries"" ADJ6 ") OR ("Anomalous" ADJ6 "aortic" ADJ6 "origin" ADJ6 "coronary" ADJ6 "artery") OR ("Anomalous" ADJ6 "aortic" ADJ6 "origin" ADJ6 "left" ADJ6 "coronary" ADJ6 "artery"" ADJ6 ") OR ("Anomalous" ADJ6 "aortic" ADJ6 "origin" ADJ6 "right" ADJ6 "coronary" ADJ6 "artery") OR ("Anomalous" ADJ6 "coronary" ADJ6 "arterial") OR ("anomalous" ADJ6 "coronary" ADJ6 "arteries" ADJ6 "originating" ADJ6 "opposite" ADJ6 "sinus" ADJ6 "valsalva") OR ("Anomalous" ADJ6 "coronary" ADJ6 "arteries") OR ("Anomalous" ADJ6 "coronary" ADJ6 "artery" ADJ6 "opposite" ADJ6 "sinus" ADJ6 "Valsalva") OR ("anomalous" ADJ6 "coronary" ADJ6 "artery" ADJ6 "originating" ADJ6 "opposite" ADJ6 "sinus" ADJ6 "valsalva") OR ("Anomalous" ADJ6 "coronary" ADJ6 "artery") OR ("Anomaly" ADJ6 "aorta" ADJ6 "origin" ADJ6 "coronary" ADJ6 "arteries") OR ("Anomaly" ADJ6 "aorta" ADJ6 "origin" ADJ6 "coronary" ADJ6 "artery") OR ("Anomaly" ADJ6 "aortic" ADJ6 "origin" ADJ6 "coronary" ADJ6 "arteries"" ADJ6 ") OR ("Anomaly" ADJ6 "aortic" ADJ6 "origin" ADJ6 "coronary" ADJ6 "artery") OR ("Anomaly" ADJ6 "aortic" ADJ6 "origin" ADJ6 "left" ADJ6 "coronary" ADJ6 "artery"" ADJ6 ") OR ("Anomaly" ADJ6 "aortic" ADJ6 "origin" ADJ6 "right" ADJ6 "coronary" ADJ6 "artery") OR ("Anomaly" ADJ6 "coronary" ADJ6 "arterial") OR ("anomaly" ADJ6 "coronary" ADJ6 "arteries" ADJ6 "originating" ADJ6 "opposite" ADJ6 "sinus" ADJ6 "valsalva") OR ("Anomaly" ADJ6 "coronary" ADJ6 "arteries") OR ("Anomaly" ADJ6 "coronary" ADJ6 "artery" ADJ6 "opposite" ADJ6 "sinus" ADJ6 "Valsalva") OR ("anomaly" ADJ6 "coronary" ADJ6 "artery" ADJ6 "originating" ADJ6 "opposite" ADJ6 "sinus" ADJ6 "valsalva") OR ("Anomaly" ADJ6 "coronary" ADJ6 "artery") OR ("Coronary" ADJ6 "anomalies") OR ("Coronary" ADJ6 "anomalous") OR ("Coronary" ADJ6 "anomaly") OR ("Coronary" ADJ6 "artery" ADJ6 "anomalies") OR ("Coronary" ADJ6 "artery" ADJ6 "anomalous") OR ("Coronary" ADJ6 "artery" ADJ6 "anomaly")).ti,ab) AND ("Intramural".mp OR "Intra mural".mp OR "Intramural*".mp OR "Intra mural*".mp))

- NOT conference review.pt
- NOT (conference review or conference abstract).pt
- AND (conference abstract).pt

**Web of Science**

<http://isiknowledge.com/wos>

TS=(("Anomalous aortic origin of a coronary artery" OR "Anomalous aortic origin of coronary artery" OR "Anomalous aortic origin of coronary arteries" OR "Anomalous aortic origin of the coronary artery" OR "Anomalous aortic origin of the coronary arteries" OR "Anomalous aortic origin of the left coronary artery" OR "Anomalous aortic origin of the right coronary artery" OR "AAOCA" OR "Anomalous coronary artery from the opposite sinus of Valsalva" OR "anomalous coronary arteries originating from the opposite sinus of valsalva" OR "anomalous coronary artery originating from the opposite sinus of valsalva" OR "ACAOS" OR "Anomalous coronary artery" OR "Anomalous coronary arteries" OR "Anomalous coronary arterial" OR "Coronary anomaly" OR "Coronary anomalies" OR "Coronary anomalous" OR "Coronary artery anomaly" OR "Coronary artery anomalies" OR "Coronary artery anomalous" OR "aberrant coronary arteries" OR "aberrant coronary artery" OR "aberrant coronary" OR (("aberrant" NEAR/6 "coronary" NEAR/6 "arteries") OR ("aberrant" NEAR/6 "coronary" NEAR/6 "artery") OR ("aberrant" NEAR/6 "coronary") OR ("Anomalies" NEAR/6 "aorta" NEAR/6 "origin" NEAR/6 "coronary" NEAR/6 "arteries") OR ("Anomalies" NEAR/6 "aorta" NEAR/6 "origin" NEAR/6 "coronary" NEAR/6 "artery") OR ("Anomalies" NEAR/6 "aortic" NEAR/6 "origin" NEAR/6 "coronary" NEAR/6 "arteries"" NEAR/6 ") OR ("Anomalies" NEAR/6 "aortic" NEAR/6 "origin" NEAR/6 "coronary" NEAR/6 "artery") OR ("Anomalies" NEAR/6 "aortic" NEAR/6 "origin" NEAR/6 "left" NEAR/6 "coronary" NEAR/6 "artery"" NEAR/6 ") OR ("Anomalies" NEAR/6 "aortic" NEAR/6 "origin" NEAR/6 "right" NEAR/6 "coronary" NEAR/6 "artery") OR ("Anomalies" NEAR/6 "coronary" NEAR/6 "arterial") OR ("anomalies" NEAR/6 "coronary" NEAR/6 "arteries" NEAR/6 "originating" NEAR/6 "opposite" NEAR/6 "sinus" NEAR/6 "valsalva") OR ("Anomalies" NEAR/6 "coronary" NEAR/6 "arteries") OR ("Anomalies" NEAR/6 "coronary" NEAR/6 "artery" NEAR/6 "opposite" NEAR/6 "sinus" NEAR/6 "Valsalva") OR ("anomalies" NEAR/6 "coronary" NEAR/6 "artery" NEAR/6 "originating" NEAR/6 "opposite" NEAR/6 "sinus" NEAR/6 "valsalva") OR ("Anomalies" NEAR/6 "coronary" NEAR/6 "artery") OR ("Anomalous" NEAR/6 "aorta" NEAR/6 "origin" NEAR/6 "coronary" NEAR/6 "arteries") OR ("Anomalous" NEAR/6 "aorta" NEAR/6 "origin" NEAR/6 "coronary" NEAR/6 "artery") OR ("Anomalous" NEAR/6 "aortic" NEAR/6 "origin" NEAR/6 "coronary" NEAR/6 "arteries"" NEAR/6 ") OR ("Anomalous" NEAR/6 "aortic" NEAR/6 "origin" NEAR/6 "coronary" NEAR/6 "artery") OR ("Anomalous" NEAR/6 "aortic" NEAR/6 "origin" NEAR/6 "left" NEAR/6 "coronary" NEAR/6 "artery"" NEAR/6 ") OR ("Anomalous" NEAR/6 "aortic" NEAR/6 "origin" NEAR/6 "right" NEAR/6 "coronary" NEAR/6 "artery") OR ("Anomalous" NEAR/6 "coronary" NEAR/6 "arterial") OR ("anomalous" NEAR/6 "coronary" NEAR/6 "arteries" NEAR/6 "originating" NEAR/6 "opposite" NEAR/6 "sinus" NEAR/6 "valsalva") OR ("Anomalous" NEAR/6 "coronary" NEAR/6 "arteries") OR ("Anomalous" NEAR/6 "coronary" NEAR/6 "artery" NEAR/6 "opposite" NEAR/6 "sinus" NEAR/6 "Valsalva") OR ("anomalous" NEAR/6 "coronary" NEAR/6 "artery" NEAR/6 "originating" NEAR/6 "opposite" NEAR/6 "sinus" NEAR/6 "valsalva") OR ("Anomalous" NEAR/6 "coronary" NEAR/6 "artery") OR ("Anomaly" NEAR/6 "aorta" NEAR/6 "origin" NEAR/6 "coronary" NEAR/6 "arteries") OR ("Anomaly" NEAR/6 "aorta" NEAR/6 "origin" NEAR/6 "coronary" NEAR/6 "artery") OR ("Anomaly" NEAR/6 "aortic" NEAR/6 "origin" NEAR/6 "coronary" NEAR/6 "arteries"" NEAR/6 ") OR ("Anomaly" NEAR/6 "aortic" NEAR/6 "origin" NEAR/6 "coronary" NEAR/6 "artery") OR ("Anomaly" NEAR/6 "aortic" NEAR/6 "origin" NEAR/6 "left" NEAR/6 "coronary" NEAR/6 "artery"" NEAR/6 ") OR ("Anomaly" NEAR/6 "aortic" NEAR/6 "origin" NEAR/6 "right" NEAR/6 "coronary" NEAR/6 "artery") OR ("Anomaly" NEAR/6 "coronary" NEAR/6 "arterial") OR ("anomaly" NEAR/6 "coronary" NEAR/6 "arteries" NEAR/6 "originating" NEAR/6 "opposite" NEAR/6 "sinus" NEAR/6 "valsalva") OR ("Anomaly" NEAR/6 "coronary" NEAR/6 "arteries") OR ("Anomaly" NEAR/6 "coronary" NEAR/6 "artery" NEAR/6 "opposite" NEAR/6 "sinus" NEAR/6 "Valsalva") OR ("anomaly" NEAR/6 "coronary" NEAR/6 "artery" NEAR/6 "originating" NEAR/6 "opposite" NEAR/6 "sinus" NEAR/6 "valsalva") OR ("Anomaly" NEAR/6 "coronary" NEAR/6 "artery") OR ("Coronary" NEAR/6 "anomalies") OR ("Coronary" NEAR/6 "anomalous") OR ("Coronary" NEAR/6 "anomaly") OR ("Coronary" NEAR/6 "artery" NEAR/6 "anomalies") OR ("Coronary" NEAR/6 "artery" NEAR/6 "anomalous") OR ("Coronary" NEAR/6 "artery" NEAR/6 "anomaly"))) AND ("Intramural" OR "Intra mural" OR "Intramural*" OR "Intra mural*"))

**Cochrane**

<https://www.cochranelibrary.com/advanced-search/search-manager>

(("Anomalous aortic origin of a coronary artery" OR "Anomalous aortic origin of coronary artery" OR "Anomalous aortic origin of coronary arteries" OR "Anomalous aortic origin of the coronary artery" OR "Anomalous aortic origin of the coronary arteries" OR "Anomalous aortic origin of the left coronary artery" OR "Anomalous aortic origin of the right coronary artery" OR "AAOCA" OR "Anomalous coronary artery from the opposite sinus of Valsalva" OR "anomalous coronary arteries originating from the opposite sinus of valsalva" OR "anomalous coronary artery originating from the opposite sinus of valsalva" OR "ACAOS" OR "Anomalous coronary artery" OR "Anomalous coronary arteries" OR "Anomalous coronary arterial" OR "Coronary anomaly" OR "Coronary anomalies" OR "Coronary anomalous" OR "Coronary artery anomaly" OR "Coronary artery anomalies" OR "Coronary artery anomalous" OR "aberrant coronary arteries" OR "aberrant coronary artery" OR "aberrant coronary" OR (("aberrant" NEAR/6 "coronary" NEAR/6 "arteries") OR ("aberrant" NEAR/6 "coronary" NEAR/6 "artery") OR ("aberrant" NEAR/6 "coronary") OR ("Anomalies" NEAR/6 "aorta" NEAR/6 "origin" NEAR/6 "coronary" NEAR/6 "arteries") OR ("Anomalies" NEAR/6 "aorta" NEAR/6 "origin" NEAR/6 "coronary" NEAR/6 "artery") OR ("Anomalies" NEAR/6 "aortic" NEAR/6 "origin" NEAR/6 "coronary" NEAR/6 "arteries"" NEAR/6 ") OR ("Anomalies" NEAR/6 "aortic" NEAR/6 "origin" NEAR/6 "coronary" NEAR/6 "artery") OR ("Anomalies" NEAR/6 "aortic" NEAR/6 "origin" NEAR/6 "left" NEAR/6 "coronary" NEAR/6 "artery"" NEAR/6 ") OR ("Anomalies" NEAR/6 "aortic" NEAR/6 "origin" NEAR/6 "right" NEAR/6 "coronary" NEAR/6 "artery") OR ("Anomalies" NEAR/6 "coronary" NEAR/6 "arterial") OR ("anomalies" NEAR/6 "coronary" NEAR/6 "arteries" NEAR/6 "originating" NEAR/6 "opposite" NEAR/6 "sinus" NEAR/6 "valsalva") OR ("Anomalies" NEAR/6 "coronary" NEAR/6 "arteries") OR ("Anomalies" NEAR/6 "coronary" NEAR/6 "artery" NEAR/6 "opposite" NEAR/6 "sinus" NEAR/6 "Valsalva") OR ("anomalies" NEAR/6 "coronary" NEAR/6 "artery" NEAR/6 "originating" NEAR/6 "opposite" NEAR/6 "sinus" NEAR/6 "valsalva") OR ("Anomalies" NEAR/6 "coronary" NEAR/6 "artery") OR ("Anomalous" NEAR/6 "aorta" NEAR/6 "origin" NEAR/6 "coronary" NEAR/6 "arteries") OR ("Anomalous" NEAR/6 "aorta" NEAR/6 "origin" NEAR/6 "coronary" NEAR/6 "artery") OR ("Anomalous" NEAR/6 "aortic" NEAR/6 "origin" NEAR/6 "coronary" NEAR/6 "arteries"" NEAR/6 ") OR ("Anomalous" NEAR/6 "aortic" NEAR/6 "origin" NEAR/6 "coronary" NEAR/6 "artery") OR ("Anomalous" NEAR/6 "aortic" NEAR/6 "origin" NEAR/6 "left" NEAR/6 "coronary" NEAR/6 "artery"" NEAR/6 ") OR ("Anomalous" NEAR/6 "aortic" NEAR/6 "origin" NEAR/6 "right" NEAR/6 "coronary" NEAR/6 "artery") OR ("Anomalous" NEAR/6 "coronary" NEAR/6 "arterial") OR ("anomalous" NEAR/6 "coronary" NEAR/6 "arteries" NEAR/6 "originating" NEAR/6 "opposite" NEAR/6 "sinus" NEAR/6 "valsalva") OR ("Anomalous" NEAR/6 "coronary" NEAR/6 "arteries") OR ("Anomalous" NEAR/6 "coronary" NEAR/6 "artery" NEAR/6 "opposite" NEAR/6 "sinus" NEAR/6 "Valsalva") OR ("anomalous" NEAR/6 "coronary" NEAR/6 "artery" NEAR/6 "originating" NEAR/6 "opposite" NEAR/6 "sinus" NEAR/6 "valsalva") OR ("Anomalous" NEAR/6 "coronary" NEAR/6 "artery") OR ("Anomaly" NEAR/6 "aorta" NEAR/6 "origin" NEAR/6 "coronary" NEAR/6 "arteries") OR ("Anomaly" NEAR/6 "aorta" NEAR/6 "origin" NEAR/6 "coronary" NEAR/6 "artery") OR ("Anomaly" NEAR/6 "aortic" NEAR/6 "origin" NEAR/6 "coronary" NEAR/6 "arteries"" NEAR/6 ") OR ("Anomaly" NEAR/6 "aortic" NEAR/6 "origin" NEAR/6 "coronary" NEAR/6 "artery") OR ("Anomaly" NEAR/6 "aortic" NEAR/6 "origin" NEAR/6 "left" NEAR/6 "coronary" NEAR/6 "artery"" NEAR/6 ") OR ("Anomaly" NEAR/6 "aortic" NEAR/6 "origin" NEAR/6 "right" NEAR/6 "coronary" NEAR/6 "artery") OR ("Anomaly" NEAR/6 "coronary" NEAR/6 "arterial") OR ("anomaly" NEAR/6 "coronary" NEAR/6 "arteries" NEAR/6 "originating" NEAR/6 "opposite" NEAR/6 "sinus" NEAR/6 "valsalva") OR ("Anomaly" NEAR/6 "coronary" NEAR/6 "arteries") OR ("Anomaly" NEAR/6 "coronary" NEAR/6 "artery" NEAR/6 "opposite" NEAR/6 "sinus" NEAR/6 "Valsalva") OR ("anomaly" NEAR/6 "coronary" NEAR/6 "artery" NEAR/6 "originating" NEAR/6 "opposite" NEAR/6 "sinus" NEAR/6 "valsalva") OR ("Anomaly" NEAR/6 "coronary" NEAR/6 "artery") OR ("Coronary" NEAR/6 "anomalies") OR ("Coronary" NEAR/6 "anomalous") OR ("Coronary" NEAR/6 "anomaly") OR ("Coronary" NEAR/6 "artery" NEAR/6 "anomalies") OR ("Coronary" NEAR/6 "artery" NEAR/6 "anomalous") OR ("Coronary" NEAR/6 "artery" NEAR/6 "anomaly"))) AND ("Intramural" OR "Intra mural" OR "Intramural*" OR "Intra mural*")):ti,ab,kw

(conference abstract OR meeting abstract OR conference proceeding OR conference proceedings):pt

**Emcare** <http://ovidsp.ovid.com/ovidweb.cgi?T=JS&NEWS=n&CSC=Y&PAGE=main&D=emcr>

(("Anomalous aortic origin of a coronary artery".mp OR "Anomalous aortic origin of coronary artery".mp OR "Anomalous aortic origin of coronary arteries".mp OR "Anomalous aortic origin of the coronary artery" .mp OR "Anomalous aortic origin of the coronary arteries".mp OR "Anomalous aortic origin of the left coronary artery" .mp OR "Anomalous aortic origin of the right coronary artery".mp OR "AAOCA".mp OR "Anomalous coronary artery from the opposite sinus of Valsalva".mp OR "anomalous coronary arteries originating from the opposite sinus of valsalva".mp OR "anomalous coronary artery originating from the opposite sinus of valsalva".mp OR "ACAOS".mp OR "Anomalous coronary artery".mp OR "Anomalous coronary arteries".mp OR "Anomalous coronary arterial".mp OR "Coronary anomaly".mp OR "Coronary anomalies".mp OR "Coronary anomalous".mp OR "Coronary artery anomaly".mp OR "Coronary artery anomalies".mp OR "Coronary artery anomalous".mp OR "aberrant coronary arteries".mp OR "aberrant coronary artery".mp OR "aberrant coronary".mp OR "Coronary Artery Anomaly"/ OR (("aberrant" ADJ6 "coronary" ADJ6 "arteries") OR ("aberrant" ADJ6 "coronary" ADJ6 "artery") OR ("aberrant" ADJ6 "coronary") OR ("Anomalies" ADJ6 "aorta" ADJ6 "origin" ADJ6 "coronary" ADJ6 "arteries") OR ("Anomalies" ADJ6 "aorta" ADJ6 "origin" ADJ6 "coronary" ADJ6 "artery") OR ("Anomalies" ADJ6 "aortic" ADJ6 "origin" ADJ6 "coronary" ADJ6 "arteries"" ADJ6 ") OR ("Anomalies" ADJ6 "aortic" ADJ6 "origin" ADJ6 "coronary" ADJ6 "artery") OR ("Anomalies" ADJ6 "aortic" ADJ6 "origin" ADJ6 "left" ADJ6 "coronary" ADJ6 "artery"" ADJ6 ") OR ("Anomalies" ADJ6 "aortic" ADJ6 "origin" ADJ6 "right" ADJ6 "coronary" ADJ6 "artery") OR ("Anomalies" ADJ6 "coronary" ADJ6 "arterial") OR ("anomalies" ADJ6 "coronary" ADJ6 "arteries" ADJ6 "originating" ADJ6 "opposite" ADJ6 "sinus" ADJ6 "valsalva") OR ("Anomalies" ADJ6 "coronary" ADJ6 "arteries") OR ("Anomalies" ADJ6 "coronary" ADJ6 "artery" ADJ6 "opposite" ADJ6 "sinus" ADJ6 "Valsalva") OR ("anomalies" ADJ6 "coronary" ADJ6 "artery" ADJ6 "originating" ADJ6 "opposite" ADJ6 "sinus" ADJ6 "valsalva") OR ("Anomalies" ADJ6 "coronary" ADJ6 "artery") OR ("Anomalous" ADJ6 "aorta" ADJ6 "origin" ADJ6 "coronary" ADJ6 "arteries") OR ("Anomalous" ADJ6 "aorta" ADJ6 "origin" ADJ6 "coronary" ADJ6 "artery") OR ("Anomalous" ADJ6 "aortic" ADJ6 "origin" ADJ6 "coronary" ADJ6 "arteries"" ADJ6 ") OR ("Anomalous" ADJ6 "aortic" ADJ6 "origin" ADJ6 "coronary" ADJ6 "artery") OR ("Anomalous" ADJ6 "aortic" ADJ6 "origin" ADJ6 "left" ADJ6 "coronary" ADJ6 "artery"" ADJ6 ") OR ("Anomalous" ADJ6 "aortic" ADJ6 "origin" ADJ6 "right" ADJ6 "coronary" ADJ6 "artery") OR ("Anomalous" ADJ6 "coronary" ADJ6 "arterial") OR ("anomalous" ADJ6 "coronary" ADJ6 "arteries" ADJ6 "originating" ADJ6 "opposite" ADJ6 "sinus" ADJ6 "valsalva") OR ("Anomalous" ADJ6 "coronary" ADJ6 "arteries") OR ("Anomalous" ADJ6 "coronary" ADJ6 "artery" ADJ6 "opposite" ADJ6 "sinus" ADJ6 "Valsalva") OR ("anomalous" ADJ6 "coronary" ADJ6 "artery" ADJ6 "originating" ADJ6 "opposite" ADJ6 "sinus" ADJ6 "valsalva") OR ("Anomalous" ADJ6 "coronary" ADJ6 "artery") OR ("Anomaly" ADJ6 "aorta" ADJ6 "origin" ADJ6 "coronary" ADJ6 "arteries") OR ("Anomaly" ADJ6 "aorta" ADJ6 "origin" ADJ6 "coronary" ADJ6 "artery") OR ("Anomaly" ADJ6 "aortic" ADJ6 "origin" ADJ6 "coronary" ADJ6 "arteries"" ADJ6 ") OR ("Anomaly" ADJ6 "aortic" ADJ6 "origin" ADJ6 "coronary" ADJ6 "artery") OR ("Anomaly" ADJ6 "aortic" ADJ6 "origin" ADJ6 "left" ADJ6 "coronary" ADJ6 "artery"" ADJ6 ") OR ("Anomaly" ADJ6 "aortic" ADJ6 "origin" ADJ6 "right" ADJ6 "coronary" ADJ6 "artery") OR ("Anomaly" ADJ6 "coronary" ADJ6 "arterial") OR ("anomaly" ADJ6 "coronary" ADJ6 "arteries" ADJ6 "originating" ADJ6 "opposite" ADJ6 "sinus" ADJ6 "valsalva") OR ("Anomaly" ADJ6 "coronary" ADJ6 "arteries") OR ("Anomaly" ADJ6 "coronary" ADJ6 "artery" ADJ6 "opposite" ADJ6 "sinus" ADJ6 "Valsalva") OR ("anomaly" ADJ6 "coronary" ADJ6 "artery" ADJ6 "originating" ADJ6 "opposite" ADJ6 "sinus" ADJ6 "valsalva") OR ("Anomaly" ADJ6 "coronary" ADJ6 "artery") OR ("Coronary" ADJ6 "anomalies") OR ("Coronary" ADJ6 "anomalous") OR ("Coronary" ADJ6 "anomaly") OR ("Coronary" ADJ6 "artery" ADJ6 "anomalies") OR ("Coronary" ADJ6 "artery" ADJ6 "anomalous") OR ("Coronary" ADJ6 "artery" ADJ6 "anomaly")).ti,ab) AND ("Intramural".mp OR "Intra mural".mp OR "Intramural*".mp OR "Intra mural*".mp))

**Academic Search Premier**

<http://search.ebscohost.com/login.aspx?authtype=ip,uid&profile=lumc&defaultdb=aph>

TX(("Anomalous aortic origin of a coronary artery" OR "Anomalous aortic origin of coronary artery" OR "Anomalous aortic origin of coronary arteries" OR "Anomalous aortic origin of the coronary artery" OR "Anomalous aortic origin of the coronary arteries" OR "Anomalous aortic origin of the left coronary artery" OR "Anomalous aortic origin of the right coronary artery" OR "AAOCA" OR "Anomalous coronary artery from the opposite sinus of Valsalva" OR "anomalous coronary arteries originating from the opposite sinus of valsalva" OR "anomalous coronary artery originating from the opposite sinus of valsalva" OR "ACAOS" OR "Anomalous coronary artery" OR "Anomalous coronary arteries" OR "Anomalous coronary arterial" OR "Coronary anomaly" OR "Coronary anomalies" OR "Coronary anomalous" OR "Coronary artery anomaly" OR "Coronary artery anomalies" OR "Coronary artery anomalous" OR "aberrant coronary arteries" OR "aberrant coronary artery" OR "aberrant coronary" OR (("aberrant" N6 "coronary" N6 "arteries") OR ("aberrant" N6 "coronary" N6 "artery") OR ("aberrant" N6 "coronary") OR ("Anomalies" N6 "aorta" N6 "origin" N6 "coronary" N6 "arteries") OR ("Anomalies" N6 "aorta" N6 "origin" N6 "coronary" N6 "artery") OR ("Anomalies" N6 "aortic" N6 "origin" N6 "coronary" N6 "arteries"" N6 ") OR ("Anomalies" N6 "aortic" N6 "origin" N6 "coronary" N6 "artery") OR ("Anomalies" N6 "aortic" N6 "origin" N6 "left" N6 "coronary" N6 "artery"" N6 ") OR ("Anomalies" N6 "aortic" N6 "origin" N6 "right" N6 "coronary" N6 "artery") OR ("Anomalies" N6 "coronary" N6 "arterial") OR ("anomalies" N6 "coronary" N6 "arteries" N6 "originating" N6 "opposite" N6 "sinus" N6 "valsalva") OR ("Anomalies" N6 "coronary" N6 "arteries") OR ("Anomalies" N6 "coronary" N6 "artery" N6 "opposite" N6 "sinus" N6 "Valsalva") OR ("anomalies" N6 "coronary" N6 "artery" N6 "originating" N6 "opposite" N6 "sinus" N6 "valsalva") OR ("Anomalies" N6 "coronary" N6 "artery") OR ("Anomalous" N6 "aorta" N6 "origin" N6 "coronary" N6 "arteries") OR ("Anomalous" N6 "aorta" N6 "origin" N6 "coronary" N6 "artery") OR ("Anomalous" N6 "aortic" N6 "origin" N6 "coronary" N6 "arteries"" N6 ") OR ("Anomalous" N6 "aortic" N6 "origin" N6 "coronary" N6 "artery") OR ("Anomalous" N6 "aortic" N6 "origin" N6 "left" N6 "coronary" N6 "artery"" N6 ") OR ("Anomalous" N6 "aortic" N6 "origin" N6 "right" N6 "coronary" N6 "artery") OR ("Anomalous" N6 "coronary" N6 "arterial") OR ("anomalous" N6 "coronary" N6 "arteries" N6 "originating" N6 "opposite" N6 "sinus" N6 "valsalva") OR ("Anomalous" N6 "coronary" N6 "arteries") OR ("Anomalous" N6 "coronary" N6 "artery" N6 "opposite" N6 "sinus" N6 "Valsalva") OR ("anomalous" N6 "coronary" N6 "artery" N6 "originating" N6 "opposite" N6 "sinus" N6 "valsalva") OR ("Anomalous" N6 "coronary" N6 "artery") OR ("Anomaly" N6 "aorta" N6 "origin" N6 "coronary" N6 "arteries") OR ("Anomaly" N6 "aorta" N6 "origin" N6 "coronary" N6 "artery") OR ("Anomaly" N6 "aortic" N6 "origin" N6 "coronary" N6 "arteries"" N6 ") OR ("Anomaly" N6 "aortic" N6 "origin" N6 "coronary" N6 "artery") OR ("Anomaly" N6 "aortic" N6 "origin" N6 "left" N6 "coronary" N6 "artery"" N6 ") OR ("Anomaly" N6 "aortic" N6 "origin" N6 "right" N6 "coronary" N6 "artery") OR ("Anomaly" N6 "coronary" N6 "arterial") OR ("anomaly" N6 "coronary" N6 "arteries" N6 "originating" N6 "opposite" N6 "sinus" N6 "valsalva") OR ("Anomaly" N6 "coronary" N6 "arteries") OR ("Anomaly" N6 "coronary" N6 "artery" N6 "opposite" N6 "sinus" N6 "Valsalva") OR ("anomaly" N6 "coronary" N6 "artery" N6 "originating" N6 "opposite" N6 "sinus" N6 "valsalva") OR ("Anomaly" N6 "coronary" N6 "artery") OR ("Coronary" N6 "anomalies") OR ("Coronary" N6 "anomalous") OR ("Coronary" N6 "anomaly") OR ("Coronary" N6 "artery" N6 "anomalies") OR ("Coronary" N6 "artery" N6 "anomalous") OR ("Coronary" N6 "artery" N6 "anomaly"))) AND ("Intramural" OR "Intra mural" OR "Intramural*" OR "Intra mural*") AND ("Tunica Media" OR "Tunica media*" OR "Arterial Media" OR "Vascular Media" OR "Venous Media" OR "Tunica" OR "Tunica*" **OR "Vascular Smooth Muscle" OR "Vascular Smooth Muscles"** OR "Adventitia" OR "Tunica adventitia" OR "Adventitial Tissue" OR "Adventitial Tissues" OR "Adventitious Tissue" OR "Adventitious Tissues" OR "Tunica Externa" OR "Adventitia" OR "Adventiti*" OR "Periadventitia" OR "Periadventiti*")) **OR** TI(("Anomalous aortic origin of a coronary artery" OR "Anomalous aortic origin of coronary artery" OR "Anomalous aortic origin of coronary arteries" OR "Anomalous aortic origin of the coronary artery" OR "Anomalous aortic origin of the coronary arteries" OR "Anomalous aortic origin of the left coronary artery" OR "Anomalous aortic origin of the right coronary artery" OR "AAOCA" OR "Anomalous coronary artery from the opposite sinus of Valsalva" OR "anomalous coronary arteries originating from the opposite sinus of valsalva" OR "anomalous coronary artery originating from the opposite sinus of valsalva" OR "ACAOS" OR "Anomalous coronary artery" OR "Anomalous coronary arteries" OR "Anomalous coronary arterial" OR "Coronary anomaly" OR "Coronary anomalies" OR "Coronary anomalous" OR "Coronary artery anomaly" OR "Coronary artery anomalies" OR "Coronary artery anomalous" OR "aberrant coronary arteries" OR "aberrant coronary artery" OR "aberrant coronary" OR (("aberrant" N6 "coronary" N6 "arteries") OR ("aberrant" N6 "coronary" N6 "artery") OR ("aberrant" N6 "coronary") OR ("Anomalies" N6 "aorta" N6 "origin" N6 "coronary" N6 "arteries") OR ("Anomalies" N6 "aorta" N6 "origin" N6 "coronary" N6 "artery") OR ("Anomalies" N6 "aortic" N6 "origin" N6 "coronary" N6 "arteries"" N6 ") OR ("Anomalies" N6 "aortic" N6 "origin" N6 "coronary" N6 "artery") OR ("Anomalies" N6 "aortic" N6 "origin" N6 "left" N6 "coronary" N6 "artery"" N6 ") OR ("Anomalies" N6 "aortic" N6 "origin" N6 "right" N6 "coronary" N6 "artery") OR ("Anomalies" N6 "coronary" N6 "arterial") OR ("anomalies" N6 "coronary" N6 "arteries" N6 "originating" N6 "opposite" N6 "sinus" N6 "valsalva") OR ("Anomalies" N6 "coronary" N6 "arteries") OR ("Anomalies" N6 "coronary" N6 "artery" N6 "opposite" N6 "sinus" N6 "Valsalva") OR ("anomalies" N6 "coronary" N6 "artery" N6 "originating" N6 "opposite" N6 "sinus" N6 "valsalva") OR ("Anomalies" N6 "coronary" N6 "artery") OR ("Anomalous" N6 "aorta" N6 "origin" N6 "coronary" N6 "arteries") OR ("Anomalous" N6 "aorta" N6 "origin" N6 "coronary" N6 "artery") OR ("Anomalous" N6 "aortic" N6 "origin" N6 "coronary" N6 "arteries"" N6 ") OR ("Anomalous" N6 "aortic" N6 "origin" N6 "coronary" N6 "artery") OR ("Anomalous" N6 "aortic" N6 "origin" N6 "left" N6 "coronary" N6 "artery"" N6 ") OR ("Anomalous" N6 "aortic" N6 "origin" N6 "right" N6 "coronary" N6 "artery") OR ("Anomalous" N6 "coronary" N6 "arterial") OR ("anomalous" N6 "coronary" N6 "arteries" N6 "originating" N6 "opposite" N6 "sinus" N6 "valsalva") OR ("Anomalous" N6 "coronary" N6 "arteries") OR ("Anomalous" N6 "coronary" N6 "artery" N6 "opposite" N6 "sinus" N6 "Valsalva") OR ("anomalous" N6 "coronary" N6 "artery" N6 "originating" N6 "opposite" N6 "sinus" N6 "valsalva") OR ("Anomalous" N6 "coronary" N6 "artery") OR ("Anomaly" N6 "aorta" N6 "origin" N6 "coronary" N6 "arteries") OR ("Anomaly" N6 "aorta" N6 "origin" N6 "coronary" N6 "artery") OR ("Anomaly" N6 "aortic" N6 "origin" N6 "coronary" N6 "arteries"" N6 ") OR ("Anomaly" N6 "aortic" N6 "origin" N6 "coronary" N6 "artery") OR ("Anomaly" N6 "aortic" N6 "origin" N6 "left" N6 "coronary" N6 "artery"" N6 ") OR ("Anomaly" N6 "aortic" N6 "origin" N6 "right" N6 "coronary" N6 "artery") OR ("Anomaly" N6 "coronary" N6 "arterial") OR ("anomaly" N6 "coronary" N6 "arteries" N6 "originating" N6 "opposite" N6 "sinus" N6 "valsalva") OR ("Anomaly" N6 "coronary" N6 "arteries") OR ("Anomaly" N6 "coronary" N6 "artery" N6 "opposite" N6 "sinus" N6 "Valsalva") OR ("anomaly" N6 "coronary" N6 "artery" N6 "originating" N6 "opposite" N6 "sinus" N6 "valsalva") OR ("Anomaly" N6 "coronary" N6 "artery") OR ("Coronary" N6 "anomalies") OR ("Coronary" N6 "anomalous") OR ("Coronary" N6 "anomaly") OR ("Coronary" N6 "artery" N6 "anomalies") OR ("Coronary" N6 "artery" N6 "anomalous") OR ("Coronary" N6 "artery" N6 "anomaly"))) AND ("Intramural" OR "Intra mural" OR "Intramural*" OR "Intra mural*"))

**ScienceDirect**

<https://www.sciencedirect.com/search>

TITLE-ABSTR-KEY**()**

("anomalous coronary" OR "anomalous aortic origin" OR "Coronary anomaly" OR "aberrant coronary") AND ("Intramural") AND ("Tunica" OR "Adventitia")

**Google Scholar**

<http://scholar.google.com/>

3960 – the first 40 articles:

18-12-2023: 4210 -> from 2023 : 267 -> the first 20 articles

"anomalous coronary"|"anomalous aortic origin"|"Coronary anomaly"|"Coronary anomalies"|"Coronary artery anomaly"|"Coronary artery anomalies"|"aberrant coronary" "Intramural"

202 - eerste 100

18-12-2023: 207 -> From 2023 : 14 -> the first 14 articles

"anomalous coronary"|"anomalous aortic origin"|"Coronary anomaly"|"Coronary anomalies"|"Coronary artery anomaly"|"Coronary artery anomalies"|"aberrant coronary" "Intramural" "Tunica media"|"Arterial Media"|"Vascular Media"|"Venous Media"

359 - eerste 50

18-12-2023: 442 -> From 2023 : 28 -> the first 28 articles

"anomalous coronary"|"anomalous aortic origin"|"Coronary anomaly"|"Coronary anomalies"|"Coronary artery anomaly"|"Coronary artery anomalies"|"aberrant coronary" "Intramural" "Vascular Smooth Muscle"|"Vascular Smooth Muscles"|"Adventitia"|"Tunica adventitia"|"Adventitial"|"Tunica Externa"
